# Ionic Liquid Electrolytes for Extreme Temperature Conditions: Challenges and Perspective

**DOI:** 10.1002/anie.7793957

**Published:** 2026-08-08

**Authors:** En Xie, Chengdong Liu, Xinghao Wang, Jian Wang, Zhen Chen, Huihua Li, Wenlong Cai, Hao Li, Yang Peng, Fanglin Wu, Stefano Passerini, Haolin Tang

**Affiliations:** ^1^ State Key Laboratory of Advanced Technology For Materials Synthesis and Processing Wuhan University of Technology Wuhan P. R. China; ^2^ Helmholtz Institute Ulm (HIU) Ulm Germany; ^3^ Karlsruhe Institute of Technology (KIT) Karlsruhe Germany; ^4^ Key Laboratory of Engineering Dielectric and Applications (Ministry of Education), School of Electrical and Electronic Engineering Harbin University of Science and Technology Harbin P. R. China; ^5^ College of Materials Science and Engineering Sichuan University Chengdu P. R. China; ^6^ Hubei Key Laboratory of Fuel Cells Wuhan University of Technology Wuhan P. R. China; ^7^ International Electrochemical Energy Storage Research Institute (IEES) School of Energy and Mechanical Engineering Nanjing Normal University Nanjing P. R. China; ^8^ National Energy Key Laboratory for New Hydrogen‐Ammonia Energy Technologies Foshan Xianhu Laboratory Foshan P. R. China

**Keywords:** battery, energy storage, interphase, ionic liquid electrolyte, low‐ and high‐ temperature operation

## Abstract

The increasing applications of electrochemical energy‐storage systems in transportation, aerospace, and grid applications impose stringent requirements on electrolytes capable of operating safely and efficiently across extreme temperatures. Conventional carbonate‐ or ether‐ based electrolytes suffer from performance degradation and severe safety hazards in extreme thermal environments. Ionic liquid electrolytes (ILEs), distinguished by their intrinsic nonflammability and remarkable resistance to temperature‐induced property fluctuations, are recognized as promising alternatives. Nonetheless, the development of wide‐temperature ILEs is constrained by their complex temperature‐dependent physicochemical behaviors and interfacial instabilities. In this review, the effects of temperature on the molecular configurations, physicochemical properties, and interfacial chemistry of ILEs are systematically elucidated. The challenges associated with ILEs operation under low‐ and high‐temperature conditions are subsequently delineated, with particular emphasis on recent advances in molecular design and co‐solvent strategies aimed at improving the wide‐temperature performance of ILEs. Furthermore, the interfacial chemistry and electrode compatibility of ILE‐based systems are examined to demonstrate their roles in dictating interphase stability and electrochemical durability. Integrating molecular‐level insights with macroscopic performance characteristics, this review presents a unified framework correlating the structure of ILEs with temperature‐dependent performance, providing valuable guidance for the rational design of next‐generation wide‐temperature ILEs toward high‐safety and high‐energy‐density rechargeable batteries.

## Graphical abstract

Ionic liquids (ILs) have gained great attention as safe electrolyte components in recent years. This review elucidates the temperature effects on IL‐based electrolytes from molecular configurations, physicochemical properties, and interfacial chemistry, raises the challenges and design strategies operating at low‐ and HT, providing valuable guidance for the rational design of high‐safety, wide‐temperature rechargeable batteries.

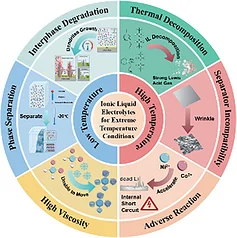

## 1 Introduction

Electrochemical energy storage technologies have gained widespread attention because of their operational simplicity, high energy‐conversion efficiency, and cost‐effectiveness [1, 2, 3]. Rechargeable secondary batteries are regarded as the leading candidates for next‐generation consumer and grid energy storage applications owing to their high energy density, stability, and portability [4, 5]. However, battery safety is the most crucial challenge for the above‐mentioned applications. Among the components of rechargeable batteries, electrolyte is regarded as playing a key role in battery safety, considering the physicochemical characteristics of conventional organic electrolytes, such as high flammability, substantial vapor pressure, and limited thermal stability, making them intrinsically susceptible to thermal runaway, gas evolution, and catastrophic failure [6, 7, 8]. The emerging solid‐state electrolytes (SSEs) propose a potential alternative; nevertheless, the challenges such as their low ionic conductivity and poor electrode‐electrolyte interface contact remain unresolved [9].

In this context, ionic liquids (ILs), characterized by non‐flammability, intrinsic thermal stability, and wide electrochemical stability windows (ESW), gained great attention and have been widely investigated as safe electrolyte components during recent years [10, 11, 12]. ILs are a class of salts composed of bulky organic cations paired with inorganic or organic anions, remaining in a liquid state at ambient temperature [13]. In contrast to traditional organic electrolytes, ionic liquid electrolytes (ILEs), composed of macroscopically homogeneous mixtures of one or more ILs and one or more salts containing electrode‐active species (e.g., Li^+^, K^+^, Na^+^, Zn^2+^), possess exceptionally high boiling points and negligible vapor pressure, endowing them with remarkable thermal robustness that mitigates temperature‐induced thermal runaway. Additionally, owing to their highly tunable molecular architectures, ILEs allow tailoring the configuration from cations and anions, or foreign co‐solvents and salts, which aids in the rational design of superior ILEs, as well as regulable solid electrolyte interphase (SEI) and cathode electrolyte interphase (CEI) layers [14, 15, 16].

In practical scenarios, battery operation environments are highly complex and diverse [17, 18, 19]. For example, the driving routes of electric vehicles may span from hot desert regions to snowy terrains (Figure 1a). Consumer electronics must withstand seasonal temperature variations across summer and winter (Figure 1b). In special cases such as spacecraft, the operating temperature of batteries can fluctuate from above 100°C to as low as −50°C within a single day (Figure 1c) [1]. Wide temperature operation requires resistance to viscosity‐driven ion transport collapse at low temperatures (LTs) and chemical stability against accelerated decomposition at high temperatures (HTs) [20]; and uncompromising safety necessitates nonflammable, thermally robust electrolytes that do not volatilize or decompose exothermically [21]. All these attributes position ILEs as promising electrolyte candidates for extreme‐temperature and safety‐critical battery applications according to the performance comparison in Figure 1d,e [22].

**FIGURE 1:**
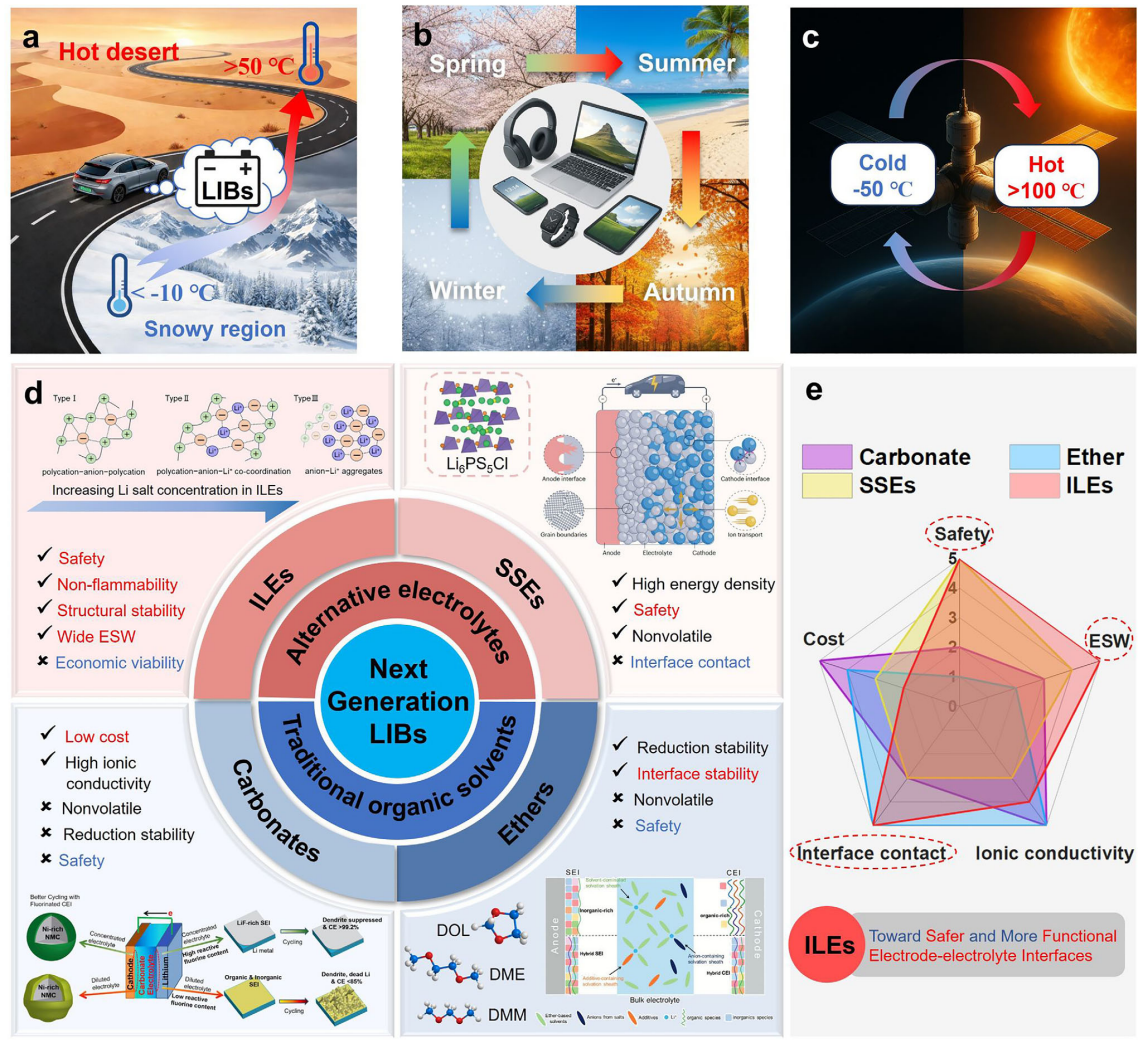
(a) Application of wide‐temperature‐range batteries in electric vehicles; (b) Operation environments for consumer electronics across four seasons; (c) Dual extreme temperatures encountered by spacecraft in space environments; (d) Comparison between conventional electrochemical systems and emerging candidates [23, 24, 25, 26]. Reprinted with permission from ref [23]. Copyright 2024 Advanced Energy Materials; Reprinted with permission from ref [24]. Copyright 2018 Chem; Reprinted with permission from ref [25]. Copyright 2024 Journal of the American Chemical Society; Reprinted with permission from ref [26]. Copyright 2025 Nature Reviews Materials; (e) Performance comparison of ILEs, ether electrolytes, carbonate electrolytes, and SSEs.

However, though the structural stability of ILEs far surpasses traditional organic electrolytes in high‐temperature environments, their practical deployment across wide temperature ranges remains far from realized [27, 28]. The highly tunable cations and anions of ILs provide great advantages for addressing the difficulty of stable operation under extreme temperatures, yet their temperature‐dependent transport constraints and interfacial instability remain key obstacles to wide‐temperature battery operation [28]. At LTs, strong electrostatic interactions lead to high viscosity and suppressed ion mobility, while solvent freezing and sluggish interfacial kinetics lead to severe polarization and capacity losses [17]. By contrast, the weakening of Coulombic interactions at HTs and reorganization of nanoscale structures can alter the solvation environments and accelerate chemical decomposition, leading to interphase destabilization and compromised separator compatibility [29]. Furthermore, temperature imposes a deeply interconnected influence on ILEs, simultaneously modifying molecular coordination motifs, Li^+^ solvation structures, and cation‐anion association, while governing the formation and dynamic reconstruction of SEI and CEI layers [20, 29]. These multiscale processes ultimately determine macroscopic outcomes including dendrite initiation, fading mechanisms, gas evolution, and battery failure [30]. Hence, elucidating the wide‐temperature behavior of ILEs necessitates a unified conceptual framework that couples molecular architecture, solvation chemistry, ion‐transport physics, and interfacial reactions under extreme temperatures. Despite growing interest in this field, existing studies typically focus on ambient temperature or isolated temperature regimes, while a comprehensive and in‐depth discussion of ILEs in extreme temperature conditions is still lacking [31, 32, 33].

Here, we fill this gap by providing a comprehensive review of ILEs from the viewpoint of temperature‐driven structure‐property‐interphase coupling. This review mainly focuses on the prospects and practical challenges of ILEs operating over a wide temperature range (from −0°C to 150°C). Although the discussion primarily focuses on the application of ILs in lithium (Li)‐based batteries, selected examples from sodium (Na)‐, potassium (K)‐, magnesium (Mg)‐, aluminum (Al)‐, and zinc (Zn)‐based batteries are also included to demonstrate the generality of the challenges and perspectives associated with ILs under extreme‐temperature conditions. We first systematically analyzed the influence of temperature on the molecular structures and physicochemical properties of ILEs, emphasized the progress and critical issues associated with their applications at LTs and HTs, and further summarized the design strategies and optimization approaches that have been proposed for improving their performance in these extreme environments. Additionally, strategies for the future development of wide temperature ILEs are proposed. This systematic analysis provides valuable insights and design guidelines for the development of battery systems capable of operating under extreme temperatures.

## 2 Mechanisms of the Temperature's Effect on ILs and ILEs

Temperature is one of the critical parameters for the properties of ILs; thus, it is essential to understand its effects from the mechanistic point of view. The temperature‐dependence of ILs’ physical and electrochemical properties originates from their unique molecular structures, which govern their thermodynamic and kinetic behaviors [34]. Within ILs, the interactions between bulky, asymmetric organic cations and weakly coordinating anions determine their bulk structure as well as the composition of the solid‐ionic liquid interphase [35]. Consequently, when the focus shifts from microscopic molecular configurations to the macroscopic physicochemical manifestations, the properties of ILs with different molecular structures exhibit distinct correlations with temperature. These temperature‐dependent properties, such as viscosity, ionic conductivity, melting point (*T*_m_), and phase‐transition behavior, exert a profound influence on the chemical reactions and associated kinetics occurring within batteries, in turn, governing the stability and safety of ILEs under HTs or subzero conditions [36, 37].

### 2.1 Characteristics of Molecular Structures

ILs represent a structurally diverse class of ion pairs whose properties can be systematically engineered through variations in cation and anion design [34]. By balancing ion‐ion interactions and molecular symmetry, ILs can access a wide range of ionic structures, while simultaneously destabilizing solid crystalline phases [13]. When temperature is regarded as a governing variable, the molecular structure of ILs fundamentally dictates the strength of Coulombic interactions, the degree of molecular asymmetry, and the extent of free volume [38]. It is therefore the structural diversity and tunability of ILs that underpin the ability to maintain relatively stable viscosity, ionic conductivity, and reaction kinetics over a wide temperature range [32]. To clarify how structural motifs control these behaviors, the following subsections examine the roles of individual cation and anion functionalities and their cooperative interactions.

#### 2.1.1 Anions

The selection of anions not only determines the ESW but also affects the *T*_m_, density, and ionic dissociation behavior of ILEs [34]. Weakly coordinating anions, like the symmetric bis(fluorosulfonyl)imide (FSI^−^) [39], bis(trifluoromethanesulfonyl)imide (TFSI^−^) [40], bis(pentafluoroethane‐sulfonyl)imide (BETI^−^) [41], as well as the asymmetric (fluorosulfonyl)(trifluoromethanesulfonyl)imide (FTFSI^−^) [42] and (nonafluorobutanesulfonyl)‐(trifluoromethanesulfonyl)imide (IM_14_^−^) [43], have been extensively employed because they effectively delocalize negative charge and reduce lattice energy. Among them, FSI^−^ generally affords higher ionic conductivity and lower viscosity due to its smaller size and greater conformational flexibility (Figure 2a). However, it exhibits lower oxidative stability at high potentials (> 4.5 V vs. Li^+^/Li) [44]. In contrast, TFSI^−^ and BETI^−^ exhibit superior thermal stability, albeit at the cost of higher viscosity and increased Li^+^ decoupling energy at subzero temperatures, which ultimately suppresses ionic transport [45, 46]. Asymmetric anions, such as FTFSI^−^ and IM_14_^−^, offer lower crystallization temperatures than their symmetric counterparts [41, 42].

**FIGURE 2:**
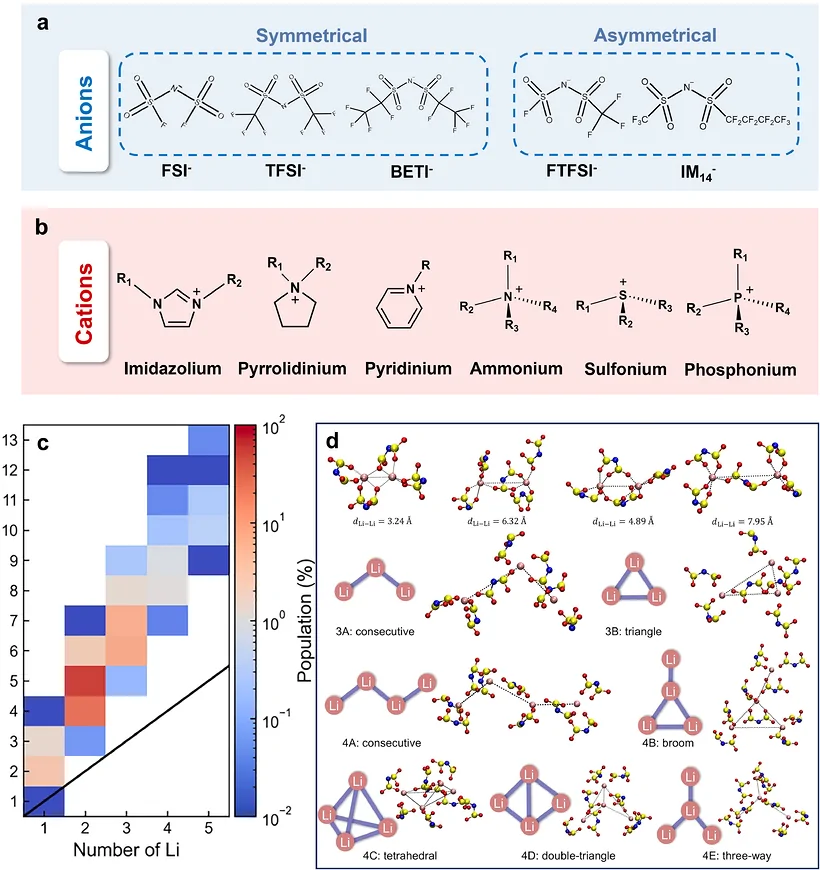
Typical cationic (a) and anionic species (b) in ILE‐based electrolytes; One of the most common cluster distributions of the electrolyte system consisting of LiTFSI and N‐butyl‐N‐methylpyrrolidinium bis(trifluoromethanesulfonyl)imide (Pyr_14_TFSI) (c) and the representative solvation structures and schematic illustrations of Li clusters (d) [47]. Reprinted with permission from ref [47]. Copyright 2025 Chemical Engineering Journal.

Moreover, the chemical softness and polarizability of anions profoundly influence ion‐pairing behavior. Hard anions (e.g., tetrafluoroborate (BF_4_^−^) and hexafluorophosphate (PF_6_^−^)) exhibit stronger electrostatic attraction toward cations, increasing crystallinity and viscosity, thereby restricting their applicability over wide temperature ranges [48]. Substituting fluorine atoms with perfluoroether groups or designing partially fluorinated anions can mitigate these effects, simultaneously achieving lower glass transition temperatures (*T*_g_) and broader ESW [48, 49].

Another effective approach for enhancing electrolyte tolerance under extreme temperatures lies in the formation of robust SEI and CEI layers [50, 51, 52]. For example, anions have been proven to play a key role in stabilizing electrode‐electrolyte interphases. Halide anions are frequently incorporated to generate uniform LiX‐rich (X = Br^−^, I^−^) SEI layers that accelerate Li^+^ interfacial transport by modulating solvation structures and facilitating desolvation processes [53]. Thus, the functional anions can effectively improve LT performance [54]. Consequently, increasing attention has shifted toward exploring functional anions, such as dicyanamide [55], sulfonate [56], and carboxylate‐based species [57], which offer improved sustainability and ionic mobility, although their long‐term electrochemical stability remains under active investigation.

#### 2.1.2 Cations

The structure of cations exerts a decisive influence on the fluidity, thermal adaptability, and electrochemical durability of ILs. Representative cationic families include pyridinium [58], imidazolium [59], pyrrolidinium [60], ammonium [61], sulfonium [62], and phosphonium derivatives [63], each imparting distinct thermal and electrochemical characteristics. Imidazolium‐based ILs with small cation size exhibit low viscosity, leading to high ionic conductivity. (Figure 2b) [64]. However, the acidic C2 proton often triggers parasitic reactions at elevated temperatures or upon contact with electrodes [65]. In contrast, saturated heterocyclic cations (e.g., pyrrolidinium) eliminate this reactive site and exhibit enhanced thermal and electrochemical stability, while showing higher viscosity. Therefore, alleviating the high viscosity of ILs composed of such cations at LTs remains a great conundrum [66].

The alkyl‐chain length and symmetry of cations also dictate temperature‐dependent behaviors [67]. Lengthening the alkyl chain weakens Coulombic interactions and disrupts lattice packing. As a result, the van der Waals forces among IL molecules become more pronounced, leading to higher viscosity and suppressing ionic mobility at LTs [34]. Introducing asymmetric or flexible side chains, such as ether, alkoxy, or fluorinated groups, can reduce lattice packing efficiency and diminish ionic association [22], which broadens the liquidus range and improves low‐temperature performance. These structural variations increase the entropic contribution to the liquid phase, allowing ILs to remain amorphous even below −40°C [68]. Furthermore, cations with larger ionic radii or multisite charge delocalization can withstand higher temperatures and minimize Coulombic binding energy with anions, resulting in greater thermal robustness. For example, ILEs containing the N‐methyl‐N‐propylpiperidinium (PP_13_) cation, TFSI^−^, and lithium bis(trifluoromethanesulfonyl)imide (LiTFSI) demonstrate remarkable thermal stability above 300°C, highlighting their promise for high‐temperature battery applications [69].

#### 2.1.3 Cation‐Anion Interactions

Beyond the individual contributions of cations or anions, the strength and spatial heterogeneity of ion‐ion interactions dictate the mesoscale organization of ILEs. Many ILEs exhibit nanostructural segregation between polar and nonpolar domains, arising from partial immiscibility between charged headgroups and hydrophobic alkyl tails [34]. Furthermore, the inherent segregation in ILEs can be critically perturbed by metal ions (e.g., Li^+^, Na^+^, K^+^, Zn^2+^ and Mg^2+^), which may ultimately induce the precipitation of specific crystalline phases at elevated concentrations. This self‐organized structure is highly temperature‐sensitive, undergoing dynamic rearrangement of ion clusters and concomitant changes in macroscopic transport properties [70].

At LTs, restricted segmental motion enhances ion pairing and reduces the fraction of mobile charge carriers [71]. However, the thermal expansion of polar domains facilitates ion diffusion decoupling at HTs. Molecular dynamics (MD) simulations demonstrate that ion correlation length and cluster lifetime vary exponentially with temperature, providing microscopic insight into the intertwined viscosity‐conductivity relationship [72]. Accordingly, rationally tuning cation‐anion pairing to incorporate structural asymmetry and charge delocalization is essential to achieve optimized interaction strength and dynamic disorder. Such molecular design ensures that ILEs maintain fluidity at subzero temperatures while preserving structural integrity under thermal stress, constituting the central design principle for developing wide‐temperature ILEs capable of supporting advanced electrochemical technologies.

### 2.2 Solvation Structures in ILs

Unlike conventional molecular solvents, ILs provide a self‐solvating medium wherein cations and anions simultaneously serve as solvent matrices and charge carriers [73]. Notably, the spatial arrangement and coordination dynamics of these ionic species around solutes directly govern critical transport properties, including ion diffusion, dissociation equilibrium, and charge‐transfer kinetics. Moreover, the temperature‐dependent evolution of solvation architectures profoundly modulates the operational thermal window of ILs [74]. Therefore, elucidating microscopic solvation structures and their thermally induced reconfiguration becomes imperative for electrolyte engineering.

#### 2.2.1 Charged Solutes in ILs

Upon dissolution of lithium or sodium salts (e.g., lithium bis(fluorosulfonyl)imide (LiFSI), LiTFSI, or sodium bis(fluorosulfonyl)imide (NaFSI)) in ILs, distinctive solvation characteristics occur compared to conventional molecular solvent‐based systems [75, 76]. In carbonate or ether‐based systems, solvent molecules with high dielectric permittivity enable well‐defined cation coordination through polar molecular interactions [77]. In contrast, ILs exhibit bidirectional solvation where both cations and anions participate, creating highly heterogeneous and spatially correlated environments [78]. Generally, steric hindrance and charge delocalization in ILs prevent direct Li‐cation coordination, resulting in anion‐dominated solvation shells that promote the formation of ion pairs and aggregates. Although this configuration enhances electrochemical stability, it concomitantly restricts ionic mobility at LTs [79].

Thermal modulation critically regulates these coordination equilibria. At lower temperatures, enhanced electrostatic interactions and attenuated thermal motion favor stabilized ionic aggregates, such as anion‐bridged Li‐rich cluster states (Figure 2c,d), where multiple Li^+^ ions are trapped within extended coordination networks mediated by TFSI^−^, thereby promoting pronounced ionic association and kinetically sluggish lithium transport [47, 80]. Conversely, elevated temperatures introduce sufficient thermal energy to disrupt these associative interactions, yielding partially dissociated mobile species that improve ionic conductivity while lowering activation energy for charge transport, although it also intensifies side reactions in cells and structural instabilities of electrodes [76]. For instance, molecular dynamics simulations demonstrate that increasing the temperature from 25°C to 100°C leads to an approximately 30% reduction in the Li^+^‐TFSI^−^ coordination number, accompanied by an increased population of free Li^+^ species [81]. This dynamical solvation reconfiguration maintains charge neutrality through temperature‐dependent coordination adjustments. The chemical identity of ILs’ components plays a crucial role in determining solvation patterns: π‐π interacting imidazolium‐based ILs form localized coordination that persists even at HTs [82], whereas aliphatic pyrrolidinium or phosphonium systems with loose ionic associations exhibit superior performance at LTs [74]. Therefore, strategic engineering of ionic architectures enables an optimized balance between coordination strength and transport flexibility, facilitating wide‐temperature operation.

#### 2.2.2 Hybrid ILE Systems and Composite Coordination Networks

While the cryogenic performance of neat ILEs remains constrained, this limitation can be mitigated through strategic formulation of hybrid ionic liquid electrolytes (HILEs) via IL blending or incorporation of molecular cosolvents [83]. These tailored electrolytes exhibit tunable coordination chemistry, synergistically integrating the complementary advantages of distinct components. For instance, combining weakly structured 1‐ethyl‐3‐methylimidazolium bis(trifluoromethanesulfonyl)imide (EMITFSI, short‐chain) with strongly structured 1‐hexyl‐3‐methylimidazolium bis(trifluoromethanesulfonyl)imide (HMIMTFSI, long‐chain) suppresses nanoscale ordering heterogeneities, thereby enabling stable operation over extended temperature ranges [84]. At the microscopic level, such mixtures generate microphase‐segregated coordination domains, where polar/apolar spatial distribution results in multiple local solvation environments [85]. This heterogeneity permits coexistence of tightly bound ionic clusters and mobile dissociated species. Upon thermal activation, the disruption of these domains facilitates accelerated ion‐exchange kinetics by lowering activation barriers. Conversely, cryogenic conditions stabilize localized domains to preserve ionic associations and suppress crystallization, thereby extending the liquidus range [86].

However, HILEs still exhibit inadequate viscosity and ionic conductivity to meet the requirements for long‐term operation under cold conditions [83]. Introduction of molecular solvents (e.g., carbonates and ethers) reshapes coordination equilibria by preferentially coordinating Li^+^, disrupting anion‐dominated solvation architectures and enhancing dissociated ion populations [87]. Nevertheless, excessive cosolvent proportion compromises intrinsic ILEs' merits such as nonvolatility and thermal resilience [88]. Therefore, identifying an optimal cosolvent ratio is crucial to achieve a trade‐off between enhanced ionic mobility and physicochemical robustness.

#### 2.2.3 Characterization Methods to Investigate the Solvation Structure in ILEs

##### 2.2.3.1 Characterization at Low Temperatures

Understanding solvation‐shell evolution at LTs is required for efficient design of ILEs. The temperature decreases can substantially alter ion coordination, cluster aggregation, and local structural heterogeneity. These microscopic changes directly affect desolvation behavior, ion transport, and interfacial kinetics, and are therefore closely related to the electrochemical failure of ILEs at LTs [89]. Accordingly, characterization methods that solve low‐temperature solvation structures need to shed light on the cause of transport restrictions in ILEs.

Variable‐temperature Raman spectroscopy is one of the most accessible and informative tools for probing low‐temperature ion association in ILs. By tracking characteristic vibrational bands of anions and cations, Raman spectroscopy can reveal changes in ion pairing, aggregate formation, and the rigidity of ionic networks as temperature decreases. In particular, the suppression of thermal broadening at LTs can improve spectral resolution and help distinguish structural features that are less evident at room temperature [89]. In this way, low‐temperature Raman analysis is highly useful for identifying the evolution of contact ion pairs, larger ionic aggregates, and local ion‐network ordering [90]. However, Raman spectroscopy is not very good at probing weak long‐range solvent‐solvent and solvent‐ion interactions, and is unable to resolve detailed solvation structures and coordination numbers [91]. Nuclear magnetic resonance (NMR) is another core technique for characterizing low‐temperature solvation structures and dynamics in ILEs. Owing to its sensitivity to local chemical environment and molecular motion, NMR can provide information on ion coordination, re‐orientational dynamics, and phase‐transition‐related structural changes [92]. But at LTs, ILs become highly viscous with longer nonequilibrium relaxation times, large spectral line widths, and reduced signal intensity, making traditional liquid‐state NMR hard to handle [93]. These difficulties can be alleviated through the use of magic‐angle spinning (MAS) in conjunction with cryogenic probes. Low‐temperature NMR can resolve chemical shift differences that are indistinguishable at room temperature, which can be used to differentiate various ion cluster configurations. For example, 2H NMR could be used to study the freezing of the reorientation motion of cations in ILs at LTs [94].

Besides spectroscopy, neutron scattering has been used to study bulk structures and dynamics, as it is sensitive to light elements (e.g. hydrogen or lithium) and has deep penetration. Small‐angle neutron scattering (SANS) is appropriate for studies of structures of 1–100 nm, and thus is appropriate to investigate ion clusters and nanoscale phase separation of ILs [95]. By isotope substitution (e.g., deuteration), the scattering contrast of different components can be tuned, which could allow a particular component to be studied (e.g., anion or cation). Cryo‐SANS experiments are typically equipped with liquid nitrogen or liquid helium cryostats, enabling real‐time monitoring of the growth or disintegration processes of ion clusters during temperature variations, thereby revealing the mechanisms of low‐temperature‐induced phase transitions.

In summary, no single characterization technique can fully resolve the multiscale solvation and clustering behaviors of ILEs under low‐temperature conditions. Raman spectroscopy is effective for tracking ion association, and NMR provides complementary insight into local coordination and dynamics. Future progress will therefore rely on the integrated use of variable‐temperature spectroscopy, scattering techniques, and theoretical simulations to achieve a complete understanding of low‐temperature solvation evolution and its correlation with ion transport and interfacial stability in ILEs.

##### 2.2.3.2 Characterization at High Temperatures

Understanding solvation‐structure evolution in HT situations will also be important to understand ILEs failure. With increasing temperatures, weak ion‐ion interactions, molecular motion, and structural relaxation may dramatically alter ion coordination, cluster distribution and inter‐temporality. In this context, variable‐temperature Raman spectroscopy and NMR remain the most commonly used experimental tools for probing HT solvation behavior, although their performance differs from that under low‐temperature conditions. In Raman spectroscopy, thermal broadening and accelerated exchange among local coordination environments often lead to broadened and weakened bands, making subtle structural distinctions increasingly difficult to resolve at elevated temperatures [96]. In contrast, moderate heating can lower viscosity and enhance molecular tumbling, leading to narrower NMR lines and improved sensitivity to coordination exchange and ion‐transport dynamics. However, high temperatures may induce decomposition or side reactions which can complicate spectral interpretation. Thus, HT NMR is valuable for monitoring dynamic interface evolution, but reliable structural assignment usually requires complementary Raman analysis, theoretical calculations, and other in situ techniques [97].

Traditional characterization techniques can be combined with molecular dynamics simulations and theoretical calculations to obtain reliable results. Compared with LT systems, the faster ionic motion at elevated temperatures shortens the characteristic timescale required to sample diffusion, ion exchange, and coordination rearrangement, which can reduce the simulation time needed to capture statistically meaningful structural evolution. Meanwhile, thermally weakened ionic interactions often smooth the local energy landscape and facilitate more direct identification of coordination‐number distributions, residence times, and temperature‐dependent cluster populations [98]. Such calculations are therefore particularly useful for quantifying the dissociation of ion aggregates, tracking the redistribution of Li^+^ solvation states, and linking microscopic structural evolution with experimentally observed changes in conductivity and interfacial stability [99]. A representative example is provided by the halozincate ILE in Zn batteries reported by Yang et al., in which MD calculations and in‐situ Raman spectroscopy revealed that the halozincate solvation structure creates a favorable coordination channel for rapid Zn^2+^ transport, thereby enabling highly reversible Zn deposition/dissolution and stable battery operation even at 80°C [100]. This finding underscores a general principle that theoretical calculations performed at elevated temperatures can directly correlate solvation‐shell reorganization with enhanced cation transport and high‐temperature electrochemical reversibility. Nevertheless, such calculations still suffer from important limitations. At HTs, the more disordered and dynamically complex nature of ILEs can increase the deviation of force‐field‐derived parameters (e.g., diffusion coefficients, coordination strength, and ion‐ion interactions) from their actual values. Furthermore, classical MD alone is not sufficient to capture bond breaking, decomposition reactions, and interface evolution, and is therefore often complemented by density functional theory calculations and continuum simulations.

### 2.3 Temperature‐Dependent Properties

Temperature fluctuations induce profound variations in viscosity, ionic conductivity, and phase transition behavior jointly influencing ion transport and electrochemical stability [34]. Elucidating these thermally responsive properties provides fundamental insights into structure‐property correlations of ILEs and guides rational electrolyte design for extreme‐condition applications.

#### 2.3.1 Viscosity and Ionic Mobility

Viscosity (η), a macroscopic indicator of intermolecular interactions, is governed by Coulombic forces and ionic network formation in ILs and ILEs [101]. At ambient conditions, strong ion‐ion correlations result in elevated viscosities [102]. With temperature rises, intensified thermal motion disrupts ionic networks, leading to exponential viscosity reduction (e.g., more than 10‐fold decrease for N‐butyl‐N‐methylpyrrolidinium bis(trifluoromethanesulfonyl)imide (Pyr_14_TFSI) when the temperature rises from 10°C to 70°C [103]). This phenomenon arises from weakened intermolecular forces and accelerated segmental dynamics, enhancing ion diffusion and reducing relaxation durations, therefore increasing ionic mobility [104]. This conclusion is further supported by several typical ILs listed in Table 1.

**TABLE 1:** Comparison of viscosity, density, ionic conductivity at 298 K and *T*_m_ of ILs composed of different anions and cations.

ILs	η (cP)	Density (g cm^−3^)	σ (mS cm^−1^)	*T*_m_ (K)	Refs.
1‐ethyl‐3‐methylimidazolium tetrafluoroborate	32	1.28	14	288	[105]
1‐ethyl‐3‐methylimidazolium bis(fluorosulfonyl)imide	34	1.5	9.2	255	[105]
1‐butyl‐3‐methylimidazolium bis((trifluoromethyl)sulfonyl)imide	51.6	1.43	4.33	268	[106]
1‐butyl‐3‐methylimidazolium tetrafluoroborate	99	1.2	3.5	192	[107]
N‐propyl‐N‐methylpyrrolidinium bis((trifluoromethyl)sulfonyl)imide	63	1.3	1.4	285	[108]
N‐butyl‐N‐methylpyrrolidinium bis((trifluoromethyl)sulfonyl)imide	85	1.4	2.2	155	[108]
butyltrimethylammomium bis((trifluoromethyl)sulfonyl)imide	116	1.4	14	280	[108]
trimethylhexylammomium bis((trifluoromethyl)sulfonyl)imide	153	—	4.3	199	[108]
1‐butyl‐3‐methylimidazolium hexafluorophosphate	274	1.37	1.5	282	[109]
1‐butyl‐3‐methylimidazolium trifluoroacetate	77	1.22	3.6	233	[109]
1‐propyl‐3‐methylimidazolium dicyanamide	70	—	10.1	—	[110]
1‐butyl‐2,3‐dimethylimidazolium dicyanamide	350	1.09	0.2	299	[110]

#### 2.3.2 Ionic Conductivity and Transport Mechanisms

Ionic conductivity (σ) exhibits an inverse correlation with viscosity according to the ideal Walden relationship, although frequently deviating from it [111]. In ILEs, the interplay among viscosity, conductivity, and temperature can be effectively described using empirical models (Figure 3a). The Arrhenius model applies when ionic motion occurs independently, with conductivity following the Arrhenius equation:

(1) $$\sigma =Ae^{-\frac{E_{a}}{RT}}$$

where, *k, E_a_, R* and *T* denote the rate constant, activation energy, universal gas constant, and the absolute temperature, respectively. However, most ILEs do not follow the Arrhenius behavior given by Equation (1) (Figure 3a), but rather Vogel–Tammann–Fulcher (VTF)‐type behavior, expressed as:

(2) $$\sigma =Ae^{-\frac{E_{a}}{R(T-T_{0})}}$$

where, *T*_0_ is the reference temperature (often close to *T*_g_). The VTF dependence underscores that ionic transport in ILEs is dynamically linked to the structural relaxation of the medium, differentiating them from ideal electrolytes where conduction is primarily governed by independent ion diffusion. This feature elucidates the significance of designing low‐viscosity ILEs or HILEs systems as a crucial method to equilibrate ionic conductivity and thermal stability over extensive temperature ranges [112].

**FIGURE 3:**
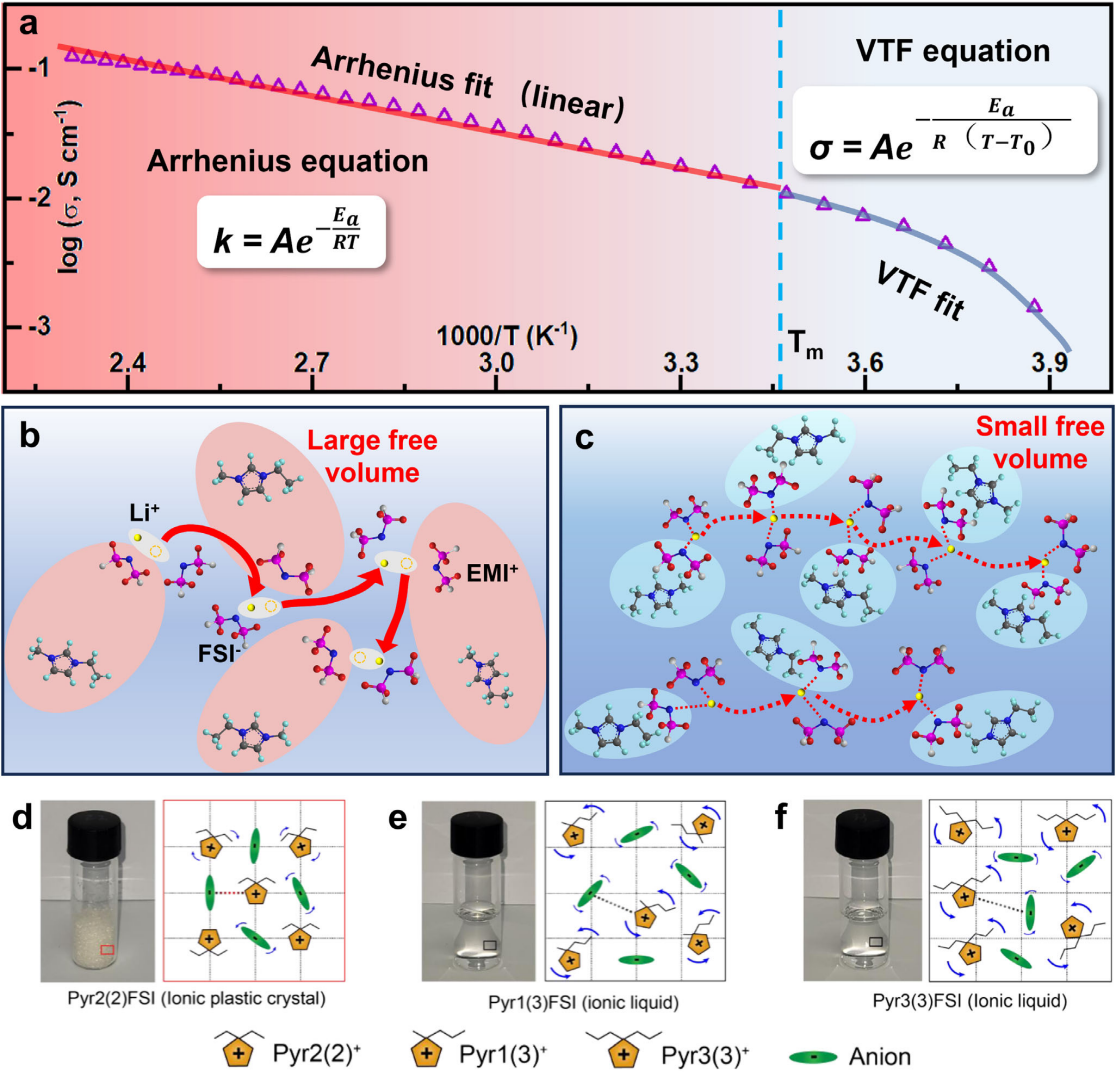
(a) σ versus temperature for 1‐ethyl‐3‐methylimidazolium tetrafluoroborate (EMIBF_4_) [113]; (b) Arrhenius behavioral model of 1‐ethyl‐3‐methylimidazolium bis(fluorosulfonyl) imide (EMIFSI) above *T*_g_; (c) VTF behavior model at LTs; photographs and schematic of microscopic structure of (d) Pyr2(2)FSI, (e) Pyr1(3)FSI, (f) Pyr3(3)FSI [114]. Reprinted with permission from ref [114]. Copyright 2025 Nature Energy.

Nonetheless, due to the existence of robust ion correlations and structural heterogeneity, actual ILEs often diverge from ideal Walden behavior since the viscosity changes according to the Arrhenius behavior (large free volume, Hopping migration) (Figure 3b) while the conductivity follows the VTF behavior (small free volume, segment‐motion‐assisted migration) (Figure 3c). At HTs, ionic conductivity markedly increases, frequently by many orders of magnitude, mainly due to improved ion mobility and diminished viscosity. This temperature relation can be elucidated through two distinct regimes: In the LT regime, ionic mobility exhibits significant correlation, and conductivity adheres to a VTF rule, signifying cooperative transport via dynamically connected ionic domains [115]. At HTs, when thermal energy surpasses the potential barriers for ion hopping, transport increasingly aligns with Arrhenius behavior, indicating a shift from “structure‐dominated” to “diffusion‐controlled” conduction. This temperature‐dependent shift directly affects electrochemical performance, particularly in LT battery applications, where elevated viscosity and slow ion transport frequently lead to diminished rate capability [116].

#### 2.3.3 Phase Transition and Glass Transition

The thermal phase behavior of ILEs encompassing *T*_m_, *T*_g_, and crystallization temperature critically determines their usability across thermal extremes. The importance of such thermal‐transition parameters has also been emphasized in sodium energy‐storage systems, where Basile et al. correlated the physicochemical and thermal characteristics of ILEs with structure‐property relationships, the effect of salt addition, and cation and anion functionalization [11]. Beyond sodium systems, Zheng et al. further summarized the applications of ILEs in potassium‐ion batteries, where electrolyte transport and interfacial kinetics become particularly sensitive to temperature because the charge carriers feature large ionic size [12]. Consequently, ion intercalation/de‐intercalation and migration during charge/discharge can be severely impeded when the electrolyte approaches *T*_g_ or undergoes crystallization, rendering electrolyte phase transitions a key bottleneck for low‐temperature operation. ILs with elongated alkyl chains or symmetric ions often show an increased tendency to crystallize, which consequently restricts ion mobility below the melting temperature [86]. On the other hand, the presence of asymmetric ions and/or large substituents leads to structural disorder, which hinders the crystallization process, giving rise to a significant supercooled liquid region characterized by a distinct glass transition [117]. At temperatures below T_g_, the movement of ions is largely restricted, resulting in very low ionic conductivity. Increasing the temperature above *T*_g_ leads to a rapid transition from glassy to liquid states, characterized by a notable increase in molecular dynamics and conductivity [86]. For instance, the symmetric 1,1‐diethylpyrrolidinium (Pyr2(2)^+^) forms a solid ionic plastic crystal with FSI^−^ at room temperature (Figure 3d), whereas ILs composed of the asymmetric 1‐methyl‐1‐propylpyrrolidinium (Pyr1(3)^+^) and the 1,1‐dipropylpyrrolidinium (Pyr3(3)^+^) with long‐alkyl‐chain paired with FSI^−^ remain liquid (Figure 3e) [114]. Additionally, various imidazolium‐based ILs exhibit *T*_g_ between −90°C and −60°C [118], while phosphonium or ammonium systems typically show higher *T*_g_ values due to stronger Coulombic interactions [119, 120].

#### 2.3.4 Molecular Descriptors of Wide‐Temperature Behavior

Viscosity, ionic conductivity, and phase‐transition behavior are typically macroscopic properties, but their temperature dependence ultimately originates from the microscopic balance among ionic interactions, local packing, and structural dynamics. This connection provides a basis for identifying a unifying descriptor to rationalize the common origins of low‐ and high‐temperature limitations in ILEs [121]. Although the challenges faced by ILEs at LTs and HTs are often discussed separately, they in fact share a common physicochemical origin: the ion‐ion interactions within ILEs cannot maintain an optimal balance for long‐term operation under extreme thermal conditions.

Free volume serves as a more relevant measure of ion‐ion interactions in ILEs, as it indicates how tightly ions are packed and how easily the ion network can be localized. A small free volume is typically associated with stronger ionic interactions and smaller local structural organization, whereas an enlarged free volume correlates with weaker structural constraints and greater configurational freedom. From this perspective, the limitations of ILEs at LTs and HTs can be understood as two opposite manifestations of free volume changes under extreme thermal conditions. At LTs, insufficient free volume leads to tighter ionic packing and strengthened Coulombic interactions, which sharply increase viscosity and impede ion transport (Figure 9c). This effect is further illustrated by the poly(1‐hexyl‐3‐vinylimidazolium)‐TFSI system, where the low surface electrostatic potential of TFSI^−^, the large side‐chain spacing of the polycation, and the weak ionic interaction energy give rise to a larger free volume, which in turn lowers the glass‐transition temperature (*T*_g_ = −66.9°C) and enhances ionic conductivity [122].

By contrast, at HTs, enlarged free volume lowers packing constraints and promotes faster ion migration, but the resulting electrolyte behavior cannot be sufficiently explained by free volume alone. Under elevated temperatures, the increased structural freedom is accompanied by more dynamic ion coordination and faster solvation‐shell reconstruction (Figure 9b). In particular, ions with stronger charge delocalization can weaken localized electrostatic binding and increase the population of weakly coordinated species. Meanwhile, the coordination number around mobile ions may fluctuate more strongly at HTs, which usually reflects the continuous breaking and reformation of local ion‐ion or ion‐solvent coordination environments [35, 123]. Although these changes can facilitate bulk ion transport, they also increase interfacial sensitivity by accelerating solvation reorganization near electrodes, triggering interphase reconstruction or thermally accelerated electrolyte degradation.

Therefore, free volume provides a useful framework for rationalizing the opposite transport and stability issues of ILEs at extreme temperatures, but it should be considered together with molecular descriptors such as ion‐ion interaction energy, charge delocalization, and coordination number. In this view, ILEs tend to become transport‐limited at LTs because of insufficient free volume and strengthened ion correlations, whereas they become increasingly interface‐ and stability‐limited at HTs because enlarged free volume is coupled with weakened coordination stability and accelerated interfacial reactions.

## 3 Challenges and Strategies of ILE at Different Temperatures

### 3.1 Low Temperatures

With decreasing temperature, the structural dynamics of ILs and ILEs progressively slow and eventually become immobilized, limiting the ability of ions and ion clusters to reorganize. This kinetic slowdown induces a dramatic rise in macroscopic viscosity and correspondingly diminishes the long‐range ionic transport. More severely, the electrolyte undergoes phase separation or crystallization, rendering ion transport pathways discontinuous and ultimately causing battery failure [22]. These LT limitations constitute a major obstacle to achieving wide‐temperature operation in energy storage systems based on ILEs. The key issues at LTs include viscosity‐driven transport suppression, crystallization‐induced phase separation, and interfacial degradation. To address these challenges, strategies comprising molecular structure design, co‐solvent engineering, additive incorporation, and interfacial regulation have been developed to enhance the LT adaptability of ILEs [124].

#### 3.1.1 Main Issues at Low Temperatures

LT conditions (typically −40°C to 0°C) remain a major bottleneck impeding practical deployment of ILEs. At such temperatures, the thermal motion of ions and solvent moieties is markedly suppressed, whereas electrostatic and van der Waals interactions are strengthened. These combined effects induce significant increase in viscosity, reduce ion mobility, elevate freezing tendencies, and heighten the likelihood of phase separation. Collectively, these LT‐induced physicochemical changes result in pronounced electrochemical degradation [124, 125, 126, 127, 128]. The following subsections delineate the primary challenges encountered at LTs and elucidate the underlying mechanisms.

##### 3.1.1.1 High Viscosity

LTs dramatically increase the viscosity of ILEs due to the diminished thermal motion of ions, which limits their ability to overcome electrostatic attractions and disrupt local ion‐pairing structures. As a consequence, rigid ionic associations and tightly bound solvation layers are formed, severely hindering ion diffusion [55]. Therefore, the temperature dependence of ion conductivity in ILEs conforms more closely to the VTF relationship under LT conditions (close to *T*_g_) [112, 129]. This behavior is strongly corroborated by experimental evidence in ILs, such as Pyr_14_‐dicyanamide (Pyr_14_DCA) and Pyr_14_‐tricyanomethanide (Pyr_14_TCM) (Figure 4a) [55]. Wang et al. demonstrated that mixed electrolytes comprising PP_13_TFSI/1,3‐dioxolane (DOL)/dimethyl‐ether (DME) (2:1:1) exhibit a sharp decline in discharge performance at LTs, which is directly attributed to markedly increased viscosity suppressing Li^+^ mobility [130]. Although sodium‐based batteries generally exhibit better LT tolerance than lithium‐based systems, the pronounced viscosity increase of ILEs at LTs remains a major obstacle to ion transport and practical electrochemical operation. Duncan et al. reported that the viscosity of borate‐containing ILEs (*N*‐ethyl‐*N,N,N*‐tris(2‐(2‐methoxyethoxy)ethyl)ammonium tetrakis(1,1,1‐3,3,3‐hexafluoroisopropoxy)borate: NaFSI = 1:1) reaches as high as 842 mPa s, virtually eliminating fluidity under LT conditions [131]. Cryogenic transmission electron microscopy (Cryo‐TEM) confirmed that high viscosity promotes heterogeneous SEI formation and elevates interfacial impedance [132]. Liu et al. further revealed a cascading failure mechanism wherein viscosity increase at LTs reduces the Li^+^ transference number, elevates the desolvation barrier, and suppresses SEI ionic conductivity, thereby positioning viscosity as the primary determinant of LT battery operability [133].

**FIGURE 4:**
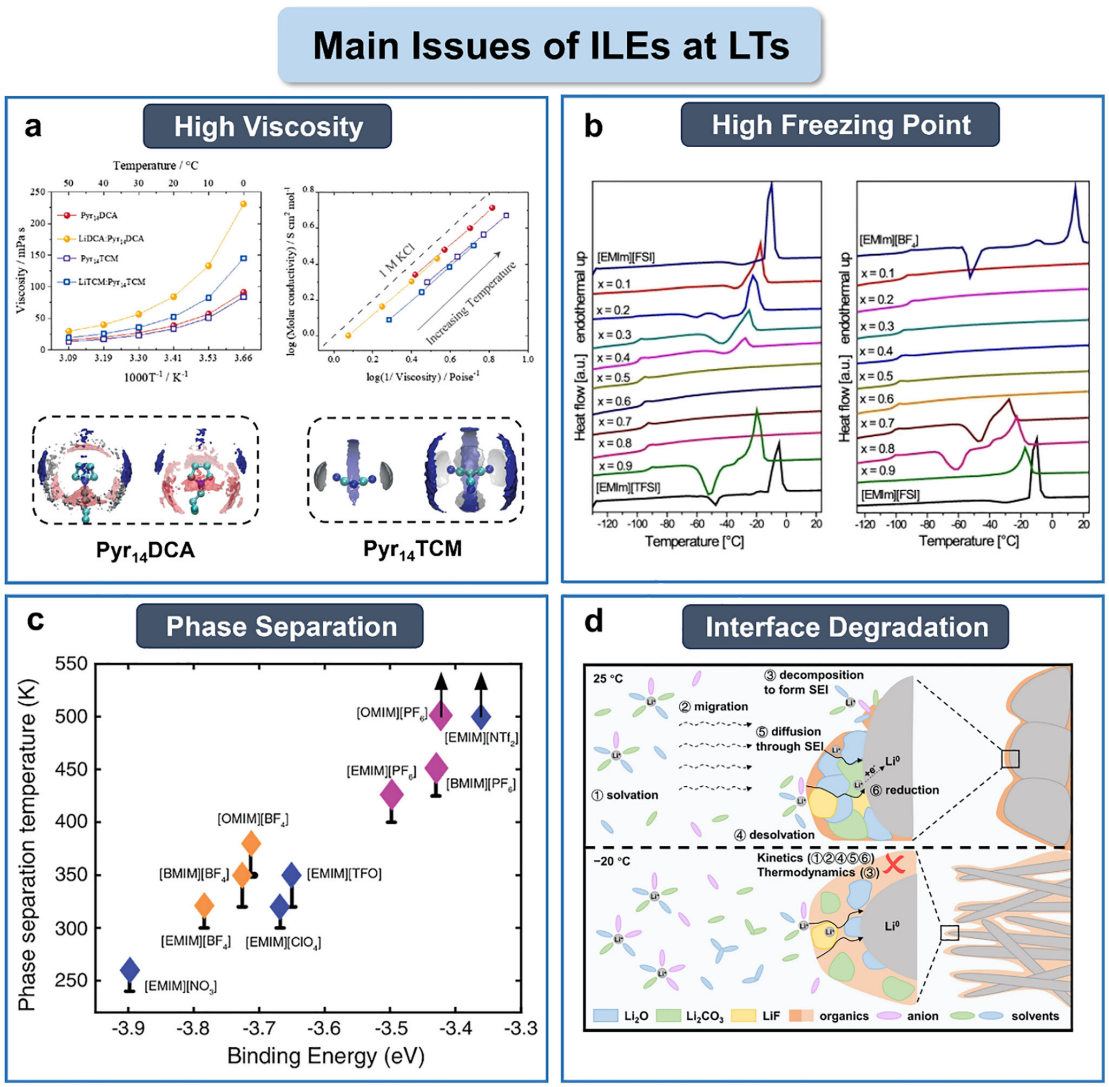
(a) Solvation shell of Pyr_14_DCA and Pyr_14_TCM, viscosity versus temperature plots of neat Pyr_14_DCA and neat Pyr_14_TCM as well as LiDCA:Pyr_14_DCA, LiTCM:Pyr_14_TCM electrolytes in 1:9 (salt: IL, mol ratio) and the corresponding Walden plot [55]. Reprinted with permission from ref [55]. Copyright 2020 Advanced Energy Materials; (b) differential scanning calorimetry (DSC) heating traces of neat ILs and their binary mixture [134]. Reprinted with permission from ref [134]. Copyright 2020 Journal of Molecular Liquids; (c) Phase separation temperature of ternary electrolytes with different ILs as a function of binding energy of IL pairs [135]. Reprinted with permission from ref [135]. Copyright 2025 Advanced Energy Materials; (d) Schematic diagram of ion diffusion and charge transfer during Li plating at room/LTs [132]. Reprinted with permission from ref [132]. Copyright 2023 Nature Communications.

##### 3.1.1.2 High Freezing Point

The inherently high freezing point of many ILs leads to crystallization or solidification at subzero temperatures, preventing ILEs from maintaining stable liquid‐phase behavior. Near *T*_m_, viscosity increases exponentially, and ionic conductivity collapses due to severely suppressed ion migration [134, 136]. For example, EMITFSI exhibits a viscosity of 175.6 mPa s at −10°C, but undergoes a rapid viscosity surge as the temperature approaches its *T*_m_ (−14°C), resulting in drastic mobility loss (Figure 4b) [134]. Freezing‐induced crystallization also destabilizes electrolyte‐electrode interphases, provoking SEI cracking or restructuring that accelerates capacity loss and shortens cycle life of cells employing ILEs [54]. At the molecular scale, freezing entails the ordering of ions from a disordered liquid into a crystalline lattice. Ions with high geometric symmetry exhibit stronger tendency toward ordered packing, and thus ILEs incorporating them possess higher T_m_. Single‐component ILs with low nucleation barriers are particularly susceptible to crystallization. Kunze et al. observed that pure Pyr_14_TFSI (*T*_m_ = −2°C) and *N*‐methyl‐*N*‐propyl pyrrolidinium (Pyr_13_FSI, *T*_m_ = −8.3°C) fully crystallize at −20°C, yielding nearly zero ionic conductivity [137]. In contrast, a binary mixture of the two ILs disrupts crystallization via size mismatch and remains liquid down to −80°C [138].

##### 3.1.1.3 Phase Separation

In multicomponent IL systems or ILEs incorporating organic co‐solvents, phase transition processes become more complex. Once phase separation occurs, electrolyte performance deteriorates sharply [139]. Such LT‐induced phase separation originates from disparities in lattice‐forming tendencies among ionic species and instability in local solvation structures (Figure 4c). The electrolyte consequently transitions from a homogeneous liquid to a heterogeneous solid‐liquid coexistence state, generating uneven ion transport pathways [135]. This heterogeneity is particularly pronounced at electrode‐electrolyte interphases, where local segregation severely disrupts charge‐transfer kinetics and accelerates performance decay. Anouti et al. demonstrated that pyrrolidinium nitrate retained strong crystallization tendencies even with co‐solvents, as indicated by multiple melting events in differential scanning calorimetry, reflecting eutectic phase separation [140]. Recently, Xie et al. employed cryo‐TEM and 4D scanning transmission electron microscopy to directly visualize nanoscale liquid‐liquid phase separation at −30°C, revealing ordered and disordered domains responsible for macroscopic conductivity deterioration [141].

##### 3.1.1.4 Degradation of Electrolyte‐electrode Interface

Under LT conditions, the degradation of the electrolyte‐electrode interface becomes a dominant contributor to the rapid degradation of cell performance. The large increase in viscosity, reduced ion mobility, and the onset of phase separation jointly hinder uniform ion flux, leading to intensified interfacial polarization and accelerating parasitic reactions [142]. On the anode side, sluggish Li^+^ transport induces inhomogeneous ion deposition and unstable SEI formation. Instead of forming a thin and uniform inorganic‐rich SEI, LT environments promote heterogeneous, organic‐rich, and resistive interphases with poor ionic conductivity. The elevated desolvation barrier further restricts Li^+^ incorporation, amplifying interfacial impedance and triggering dendritic or mossy lithium growth [143, 144]. Nevertheless, cathode interfacial degradation proceeds through distinct mechanisms. LTs significantly hinder ion (de)intercalation kinetics within layered or spinel structures, increasing overpotential and suppressing phase transitions during cycling (Figure 4d) [132]. Meanwhile, the reduced interfacial reactivity impedes the formation of a coherent CEI layer. At LTs, ILEs generate patchy, incomplete, and chemically unstable CEI layers that fail to provide sufficient protection against electrolyte oxidation or transition‐metal dissolution, thereby accelerating side reactions and impedance growth [138, 144]. Consequently, the simultaneous instability of both SEI and CEI under LT conditions establishes a direct pathway toward rapid interfacial failure and long‐term performance deterioration.

#### 3.1.2 Design Strategies

Addressing the LT limitations of ILEs requires design principles that simultaneously modulate bulk physicochemical properties, solvation structures, and interfacial reactions. Accordingly, existing approaches can be hierarchically categorized into three levels: (1) molecular engineering of ionic species to intrinsically reduce viscosity and suppress crystallization; (2) additive‐mediated regulation to construct robust SEI/CEI layers also facilitating interfacial ion transport; (3) co‐solvent or mixed IL strategies that minimize phase separation, depress the freezing point, and optimize the solvation environment. Together, these strategies form a multidimensional framework for enhancing the LT operation of ILEs.

##### 3.1.2.1 Cation/Anion Design

Molecular engineering of ionic species represents one of the most fundamental strategies for overcoming the intrinsic LT limitations of ILEs. By tailoring cation/anion structures, particularly the cation, which governs viscosity, packing behavior, and solvation strength of ILs, researchers aim to reduce lattice energy, weaken ion‐pairing interactions, increase configurational freedom, and regulate solvation environments. Three main design principles have been established: incorporation of flexible ether chains, construction of asymmetric ionic structures, and functionalization with hydroxyl or fluorinated groups. These molecular strategies fundamentally reshape the physicochemical behavior of ILEs at LTs.

Incorporation of ether chains into cations has proven highly effective in reducing viscosity and enhancing ionic mobility at LTs. Ether segments disrupt crystalline packing, expand free volume, and facilitate segmental motion [78]. In addition, the lone‐pair electrons of ether oxygen atoms offer weak Lewis basicity, enabling transient Li^+^/Na^+^ coordination that moderates strong cation‐anion interactions [145]. Consequently, ionic mobility is enhanced while providing directional solvating sites that promote ion transport. For instance, the ether‐functionalized tributyl(methoxymethyl) phosphonium hexafluorophosphate (P_4441O1_PF_6_) facilitates PF_6_^−^‐derived cluster formation via cationic shielding, while ether‐Li^+^ interactions effectively lower the viscosity of the Li salt/IL system, collectively enabling both a stabilized inorganic‐rich interphase and a favorable Li^+^ transport rate [125]. To some extent, ether‐functionalized cations not only enhance bulk ionic transport but also stabilize the electrode interface at LTs (Figure 5a,b).

**FIGURE 5:**
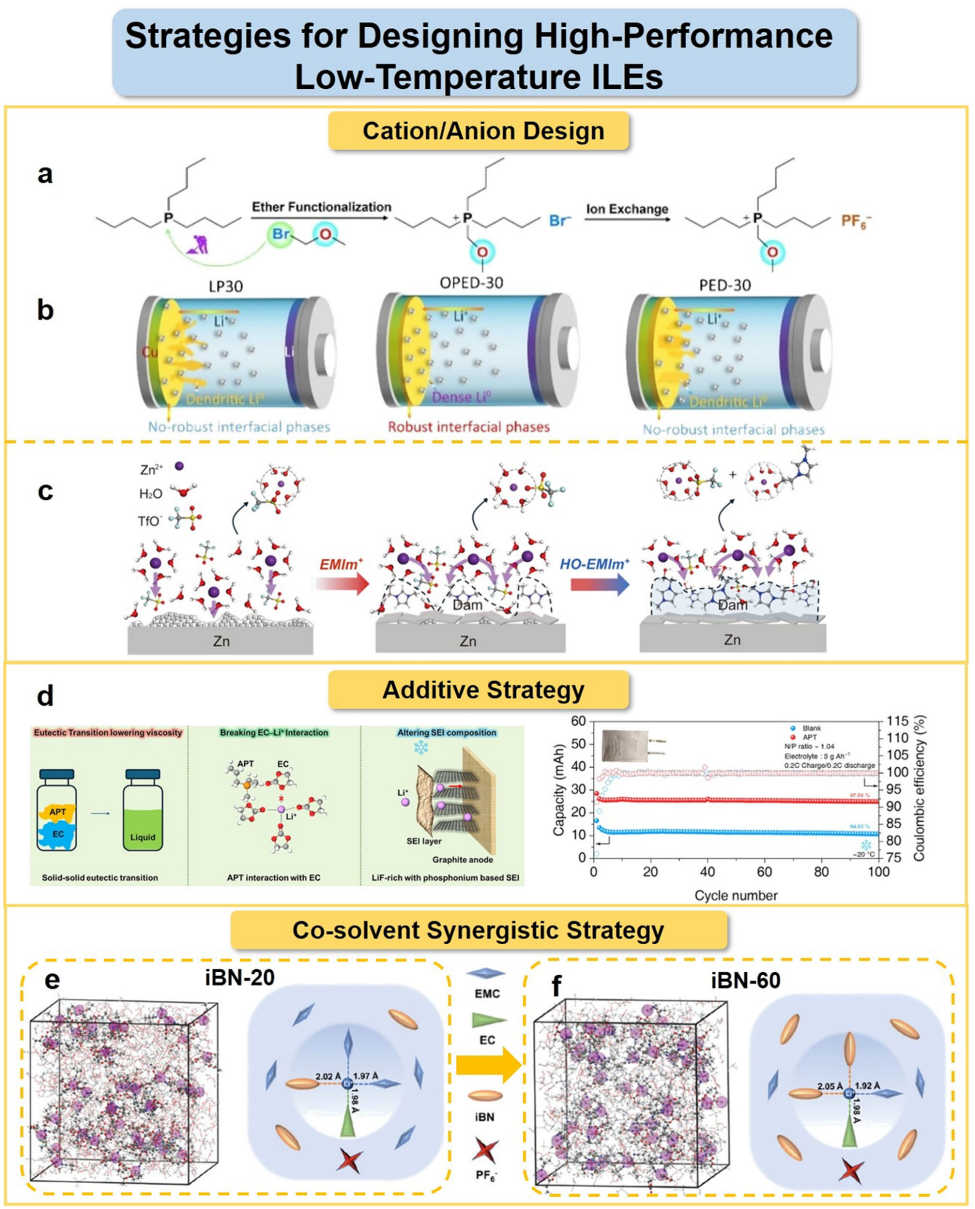
(a) The design concepts for P_4441O1_PF_6_ and (b) schematic illustration of Li deposition with LP30 (a commercial electrolyte), OPED‐30 (30 wt.% P_4441O1_PF_6_ in LP30) and PED‐30 (non‐ether‐functionalized) [125]. Reprinted with permission from ref [125]. Copyright 2025 Chemical Engineering Journal; (c) schematic illustration of Zn^2+^ solvation sheaths and Zn anode interfaces without additives (left) and with EMIM (middle) and HO‐EMIM (right) [146]. Reprinted with permission from ref [146]. Copyright 2025 Advanced Functional Materials; (d) schematic illustration of the improved subzero‐temperature performance enabled by the APT additive and cycling performance of NCM811||Gr pouch cells with blank and APT electrolyte at −20°C [147]. Reprinted with permission from ref [147]. Copyright 2025 Advanced Energy Materials; Snapshots and schematic diagrams of the solvation structures of (e) the iBN‐20 and (f) iBN‐60 electrolytes [148]. Reprinted with permission from ref [148]. Copyright 2023 Advanced Materials.

Introducing asymmetry into cations or anions disrupts molecular packing, increases configurational entropy, and inhibits crystallization, which critically influences the freezing behavior of ILEs. Early ILs often suffered from high melting temperatures arising from efficient crystal packing and strong intermolecular interactions. Subsequent molecular design strategies, exemplified by Wilkes’ introduction of the EMI^+^ cation in 1992, demonstrated that reducing packing efficiency through tailored alkyl substitution effectively lowers lattice energy and *T*_m_, laying the foundation for room‐temperature ILs [149]. Recent studies further demonstrate the effectiveness of asymmetry‐driven design; for example, asymmetric substitution on imidazolium cations has been shown to markedly reduce *T*_m_ and *T*_g_. Mac Farlane et al. reported that introducing unequal alkyl chain lengths or branching on imidazolium cations significantly disrupted ionic packing efficiency, leading to pronounced depression of crystallization and improved ionic mobility at subzero temperatures [146]. Beyond imidazolium systems, intrinsically asymmetric ammonium and phosphonium cations, particularly those incorporating ether‐functionalized side chains, have been demonstrated to further enhance configurational freedom and weaken Coulombic ordering. Michael et al. showed that ether‐containing asymmetric ammonium cations induce transient intramolecular folding and conformational disorder, effectively lowering viscosity and enabling stable liquid‐state behavior down to −20°C even when paired with structurally symmetric anions [150]. In parallel, anion‐side asymmetry has also been explored as an effective strategy to inhibit crystallization. Asymmetric sulfonate‐ or alkyl‐functionalized anions introduce uneven charge delocalization and steric frustration, which disrupt long‐range ionic ordering and suppress cooperative freezing behavior [151]. Such asymmetry‐driven disorder at the ion‐pair level has been widely recognized as a key factor in achieving low *T*_g_ and enhanced LT fluidity in IL‐based electrolytes.

Hydroxylation or fluorination of IL components provides further regulation over solvation structures and interfacial chemistry under LT conditions. Hydroxyl‐functionalized ILs form hydrogen‐bond networks that reorganize solvation shells and suppress undesirable reactions. Importantly, such molecular‐level design of IL components is not limited to lithium‐based systems, but can also be extended to other metal batteries, such as Zn batteries, where tailoring cation/anion functionality similarly regulates solvation chemistry and interfacial reactions under LT conditions. For example, Yan et al. developed 1‐hydroxyethyl‐3‐methylimidazolium triflate (HO‐EMITfO), which displaced water molecules in the Zn^2+^ solvation shell, thereby inhibiting hydrogen evolution and reducing corrosion (Figure 5c). Zn‖Zn cells with this IL cycled stably for 1800 h at 15°C, and Zn‖Cu cells maintained 99.5% Coulombic efficiency (CE) over 1000 cycles [146]. Hu et al. engineered a hydroxylated IL‐based hydrogel (PIL‐OH) capable of forming hydrogen bonds with water, disrupting the water‐water network and inhibiting ice nucleation. Aqueous lithium‐ion batteries (LIBs) using this hydrogel achieved an ionic conductivity of 0.08 mS cm^−^^1^ at −80°C and retained 93% of capacity after 200 cycles [152]. Conversely, fluorination enhances interfacial stability and is usually paired with ether‐functionalized ILs to synergistically improve ion mobility and SEI/CEI robustness [86]. Collectively, these molecular engineering strategies reduce ion‐pair binding energies, increase configurational flexibility, lower viscosity and freezing points, and stabilize interfacial layers. They therefore establish the material foundation necessary for subsequent additive and co‐solvent engineering strategies to improve LT operation of ILEs.

##### 3.1.2.2 Additive Strategy

Additives offer a versatile and powerful approach for improving LT performance of ILEs by directly regulating solvation structures and constructing robust electrode‐electrolyte interphases. Unlike molecular design strategies that modify intrinsic bulk properties, additive engineering enables dynamic tuning of interfacial chemistry, ion‐transport kinetics, and LT stability through targeted chemical pathways [153]. Additives generally fall into three functional categories: (i) film‐forming agents that generate robust lithium fluoride (LiF)‐rich SEI/CEI layers, (ii) ion‐transfer enhancers that reduce the desolvation barrier, (iii) electric‐field homogenizers that regulate electric double layer (EDL) structure and suppress interfacial hotspots. These functionalities collectively mitigate core LT bottlenecks of ILEs, including viscosity‐induced ionic mobility loss, interfacial impedance growth, dendritic deposition, and electrolyte decomposition.

Film‐forming additives are the most widely employed and operate through preferential reduction at LTs to construct inorganic‐rich, uniform SEI/CEI layers. Owing to a low lowest unoccupied molecular orbital (LUMO) energy, fluoroethylene carbonate (FEC) undergoes C‐F bond cleavage at subzero temperatures, generating LiF and forming compact SEI layers. Zhang et al. demonstrated FEC‐containing electrolytes enabling Cu‖Li cells to achieve 98% CE and LiNi_0.8_Co_0.1_Mn_0.1_O_2_ (NCM811)‖Li cells to retain 65% capacity at −20°C [154]. Song et al. introduced lithium difluorobis(oxalato) phosphate (LiDFBOP), a bifunctional additive producing Li_2_CO_3_/LiF and elastic Li_x_(FOP)_n_ frameworks upon reduction at 1.9 V, yielding thin and mechanically resilient SEI layers. Graphite‖Li cells exhibited doubled LT capacity (80 mAh g^−1^ at −20°C), while NCM‖graphite cells maintained 87% capacity over 125 cycles at −20°C [155]. Further studies showed that combining FEC with LiDFBOP resulted in ultrathin (10–20 nm), high‐modulus SEI layers that remain stable from −95°C to +70°C, enabling LiNi_0.8_Co_0.15_Al_0.05_O_2_‖Li cells to deliver 96 mAh g^−1^ even at −85°C [50]. These studies demonstrate that film‐forming additives operate via a sequential mechanism, involving preferential reduction, inorganic framework formation, and organic/inorganic filling, which produces highly resilient interfacial protection. Ion‐transfer enhancers improve LT operation by regulating the solvation structure and lowering Li^+^ desolvation energy. These additives typically engage in weak, rapidly exchanging ion interactions, preserving favorable contact ion pairs (CIP) ratio while preventing excessive aggregate ion pairs (AGG) formation. Zhang et al. developed a localized high‐concentration electrolyte (LHCE) system using acetonitrile (AN) and 1,1,2,2‐tetrafluoroethyl 2,2,2‐trifluoroethyl ether (TTE) as a weakly coordinating diluent of LiFSI, effectively preventing Li^+^‐FSI^−^/AN cluster aggregation at ‐20°C and reducing Li^+^ desolvation energy from 53.5 to 31.5 kJ mol^−1^, enabling graphite electrodes to retain 264 mAh g^−1^ at −20°C (0.1C) [156]. A similar solvation‐regulation strategy has also been shown to be effective in Na‐based battery systems, Pai and Manthiram demonstrated that cyclopentyl methyl ether (CPME) promoted a favorable Na^+^‐FSI^−^ CIP/AGG ratio, yielding Na‖Na cells with only 50 mV overpotential for 2500 h and Na‐sulfurized polyacrylonitrile cells retaining 720 mAh g^−^^1^ after 300 cycles [157]. Similar enhancements were observed in deep eutectic solvents, where propylene carbonate (PC) generates “spatial isolation layers” enhancing anion diffusion and cathode recyclability [158]. These findings elucidate that ion‐transfer enhancers mitigate viscosity‐induced suppression by moderating ion coordination and accelerating desolvation kinetics.

Electric‐field homogenizers offer a complementary mechanism of improving LT cycling stability by regulating EDL structure and reducing local interfacial field gradients [158]. Small anions and non‐ionic surfactants homogenize surface charge and suppress dendritic nucleation. Wang et al. showed that Cl^−^ anions generate compact EDL that stabilize Li_x_PF_y_O_z_‐rich SEI layers, significantly reduce electrolyte decomposition, and enable 92.3% capacity retention and 99.8% CE [159]. A multifunctional additive, allyl trimethyl phosphonium bis(trifluoromethanesulfonyl)imide (APT), can weakens Li^+^‐solvent interaction, lowers viscosity, improves LT ionic conductivity and promotes thin, LiF‐rich SEI formation (Figure 5d). With only 1 wt.% APT, high‐loading NCM811||graphite pouch cells retain 87.56% capacity at −20°C after 100 cycles, far exceeding additive‐free cells (64.60%) [147]. These findings demonstrate that field‐homogenizing additives effectively mitigate LT interfacial failure modes by suppressing hotspots and promoting uniform deposition.

##### 3.1.2.3 Multi‐Solvent Combination

Although single‐component additives can effectively address specific limitations of ILEs, they rarely overcome the combined challenges of viscosity‐governed transport, freezing‐induced phase transitions, and interfacial instability at LTs. Therefore, co‐solvent synergistic strategies have emerged as highly effective approaches that leverage complementary physicochemical properties of ILs, co‐solvents, and salts [22, 86]. Their evolution can be divided into three stages: early dilution, functional co‐solvent engineering, and molecular‐level integrated design.

Early efforts primarily focused on diluting viscous ILs with low‐viscosity, low‐melting organic solvents to expand the liquid‐phase window and enhance ion transport. Classic co‐solvents included carbonates, acrylates, and esters. Yoshizawa et al. demonstrated that mixing EMITFSI with poly(ethyleneoxide) increased conductivity by reducing the density of ion pairs. Bansal et al. revealed that incorporating ethylene carbonate (EC)/PC and 1‐ethyl‐3‐methylimidazolium bisperfluoroethylsulfonyl imide into poly(vinylidene fluoride‐co‐hexafluoropropylene) (PVDF‐HFP) copolymer gel enabled VTF‐type conduction behavior with a low *T*_m_ (−57.5°C), suppressing LT glass transition [129]. However, these systems suffered from limited thermal stability, narrow ESW, volatile co‐solvents compromising IL safety, and unstable SEI formation.

Functional co‐solvent engineering subsequently shifted focus from simple viscosity reduction to targeted modulation of solvation and interfacial regulation. Fluorinated carbonates such as FEC gain an important role due to their dual functions in reducing viscosity and forming LiF‐rich SEI layers. Even at low concentrations, FEC enhances interfacial stability, as demonstrated by Zhang et al., who reported significant increases in CE and cycle life in LiTFSI/N‐methyl‐N‐allylpyrrolidinium bis(trifluoromethanesulfonyl)imide/ LiPF_6_/EC/PC/ethyl methyl carbonate (EMC) systems [160]. Fluorinated ethers (e.g., TTE, ethyl 1,1,2,2‐tetrafluoroethyl ether) further extended oxidation stability of LiTFSI/Pyr_13_FSI electrolyte, enabling robust CEI formation at high voltage, while fluorinated aromatics such as 1,2‐difluorobenzene facilitated dense LiF‐rich SEI layers that inhibited dendrite growth [161]. Despite their advantages, functional co‐solvents introduce challenges such as high costs, environmental concerns, and complex viscosity‐stability trade‐offs, particularly at high concentrations.

Recent developments have shifted toward molecular‐level integrated design, where ILs, co‐solvents, and salts form cooperative solvation structures optimized for both bulk transport and interfacial stability. In this context, LHCEs have been recognized as an important design framework for achieving multifunctional electrolytes with improved interfacial compatibility, non‐flammability, and LT adaptability [162]. LHCEs represent a key embodiment of this concept. Chen et al. identified donor number (DN ≤ 10) as the key descriptor for co‐solvents that preserve the ILs‐rich network structure [163]. Low‐DN co‐solvents such as tetrahydrofuran induce IL‐anion aggregation into nanoscale domains that enhance Li^+^ mobility while maintaining non‐flammability. Ueno et al. demonstrated that nonpolar highly concentrated electrolytes (HCEs) generate bicontinuous IL‐rich and solvent‐rich domains where ILs microphases provide transport pathways and nonpolar solvents reduce viscosity [164]. Wu et al. further observed heterogeneous double layers in ILs with co‐solvent systems, in which IL‐rich domains facilitate LiF‐rich SEI formation while co‐solvent‐rich domains promote favorable solvation energetics [165]. Moreover, incorporating weakly solvating, low‐viscosity isobutyronitrile (iBN) effectively moderated Li^+^ affinity, enabling IL‐based electrolytes to maintain an ionic conductivity of 1.152 mS cm^−^^1^ at −70°C. Under this ultralow condition, LiCoO_2_||graphite pouch cells retain 68.7% of their room‐temperature capacity and cycle stably from −40°C to 60°C (Figure 5e,f) [148].

These integrated systems offer the most balanced and effective strategies for addressing both freezing and interfacial stability. However, their structural complexity, increased costs, and challenges in mechanistic characterization hinder large‐scale commercialization. Advanced spectroscopic characterizations and simulation techniques are required to elucidate the multi‐component interactions that underpin their performance. In summary, mixing ILs and co‐solvent strategies have evolved from simple viscosity reduction to a sophisticated, synergistic design framework. By leveraging the interactions among ILs, co‐solvents, and salts, this approach offers a versatile pathway for achieving wide‐temperature operability in next‐generation ILE systems.

To facilitate comparison among the major LT optimization strategies for ILEs, their main advantages and limitations are briefly summarized in Table 2. Overall, molecular design fundamentally determines the intrinsic physicochemical properties; additive strategies primarily optimize interfacial stability; while multi‐solvent approaches provide the most comprehensive solution by simultaneously addressing transport, phase behavior, and interfacial challenges at LTs.

**TABLE 2:** Comparative analysis of strategies for improving LT performance of ILEs.

Strategy	Subcategory	Advantages	Limitations
Molecular design	Ether‐functionalized ions	High ion mobility; stable interphase	Complex synthesis
	Asymmetric ion structures	Low melting point; low viscosity	Limited interfacial regulation
	Hydroxyl/fluorinated functionalization	Interfacial stability; improved transport	Requires combined strategies
Additive strategy	Film‐forming additives	Stable interphase; improved cycling	Stable interphase; improved cycling
	Ion‐transport enhancers	Fast ion transport; low desolvation barrier	Sensitive to formulation
	Electric‐field homogenizers	Uniform deposition; suppressed dendrites	Limited improvement of bulk‐property
Multi‐solvent strategy	Simple co‐solvent dilution	Low viscosity; high conductivity	Reduced safety; limited stability
	Functional co‐solvents	Balanced transport and stability	High cost; trade‐offs
	Integrated design	Superior LT performance	High complexity; poor scalability

### 3.2 High Temperatures

High‐temperature operation is increasingly unavoidable in practical battery environments, where local cell temperature can reach 80°C–140°C due to intensive fast‐charging, high‐rate cycling, and/or operation in thermal enclosures, and under such conditions safety and high‐temperature durability become as critical as energy and power output [21]. Such elevated conditions fundamentally reshape electrolyte stability and interfacial reaction pathways. Conventional electrolytes exhibit satisfactory performance at room temperature; however, they undergo significant degradation at elevated temperatures (typically above 80°C), thereby introducing considerable safety concerns [166]. ILEs, recognized for their distinctive thermal stability, poor flammability, and non‐volatility, are regarded as promising candidates for HT battery applications [167]. Nevertheless, their practical implementation under HT conditions presents several challenges. This section elucidates the primary challenges encountered by ILEs under thermal stress, with particular emphasis on the stringent requirements for thermal and electrochemical stability [168]. Furthermore, it provides a systematic overview of recent advances that aim to resolve these issues through rational material design, additive engineering, and interfacial optimization.

#### 3.2.1 Main Challenges at High Temperatures

Elevated temperatures impose a series of strongly coupled physicochemical perturbations on ILEs that fundamentally reshape their bulk structures, solvation environments, interfacial chemistry, and ultimately battery performance. Although some ILs exhibit intrinsically higher thermal stability and nonflammability compared with carbonate and ether‐based solvents, their HT response is governed by a complex interplay of structural reorganization and reactive decomposition rather than a simple increase in reaction kinetics.

##### 3.2.1.1 Structure Thermal Stability

At the bulk‐electrolyte level, thermal activation progressively disrupts the nano‐segregated domains and long‐range Coulombic ordering that typify many ILEs. This thermally induced reorganization modifies cation‐anion association equilibria, adjusts Li^+^ coordination numbers, and alters solvation energies, thereby reshaping dominant ion‐transport mechanisms and opening additional pathways for chemical reactions (Figure 6a). Calorimetric and thermogravimetric investigations have demonstrated that although many ILs and IL‐based electrolytes exhibit apparent thermal decomposition above 350°C [118, 169], in situ spectroscopic and analytical techniques reveal that substantial chemical decomposition, manifested as color changes and gas evolution, can already occur at temperatures as low as ∼150°C, i.e., well below the decomposition onset inferred from thermogravimetric analysis [170]. In particular, ILEs incorporating fluorinated anions such as FSI^−^ and TFSI^−^, which are widely employed in high‐temperature pyrrolidinium‐type systems, have been shown to generate sulfur‐, nitrogen‐ and fluorine‐containing fragments under prolonged thermal exposure [171, 172, 173]. These reactive intermediates progressively alter the bulk electrolyte composition, and moreover, actively participate in subsequent interfacial reactions with electrode surfaces. Therefore, bulk thermal stability inferred from macroscopic metrics alone may substantially overestimate the true chemical resilience of ILEs under realistic high‐temperature operating conditions.

**FIGURE 6:**
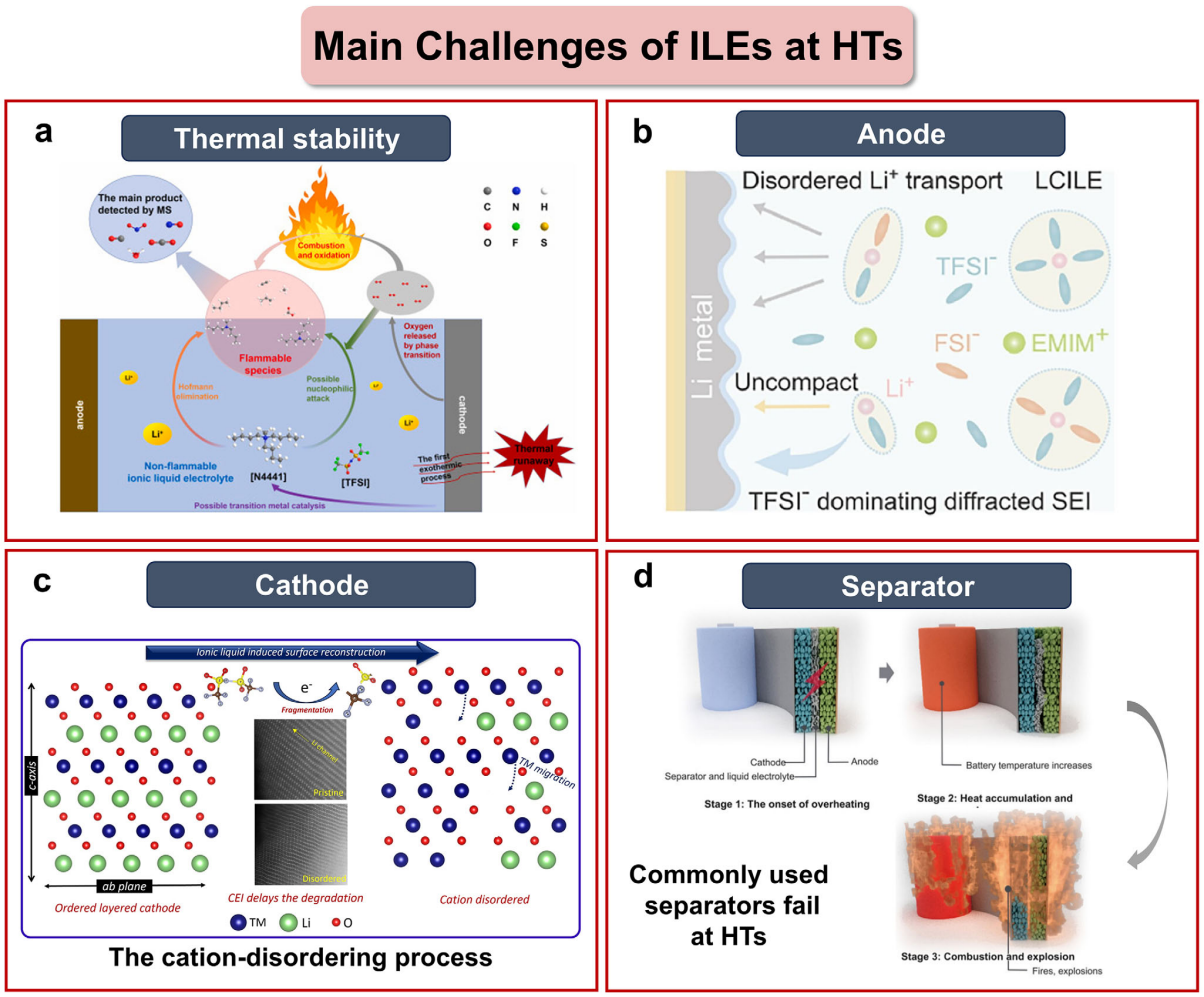
(a) Schematic description of the preliminary hypothesis of the first exothermic reaction process at 200°C–300°C for the TFSI‐based ILE in the NCM811‐silicon carbon system [174]. Reprinted with permission from ref [174]. Copyright 2025 Renewable and Sustainable Energy Reviews; (b) Schematics of Li deposition [167]. Reprinted with permission from ref [167]. Copyright 2024 Energy Storage Materials; c) Schematic representation of the formation of surface reconstruction layer on cathode [175]. Reprinted with permission from ref [175]. Copyright 2025 Cell Reports Physical Science; (d) Three stages for LIBs thermal runaway process [176]. Reprinted with permission from ref [176]. Copyright 2025 Energy Storage Materials.

##### 3.2.1.2 Interphase Stability

Interphase stability constitutes a second, but equally critical challenge at elevated temperatures. The SEI and CEI layers that remain reasonably robust at moderate temperatures frequently undergo thermal softening, structural reconstruction, and partial dissolution as cell temperature rises. Consequently, fresh lithium metal surfaces are continuously re‐exposed to the electrolyte, triggering renewed parasitic reactions and amplifying electrochemical cross‐talk between the electrodes. Experimental evidence demonstrates that ILEs, despite their intrinsic non‐volatility, can still engage in accelerated interfacial reactions at elevated temperatures (Figure 6b). For example, pyrrolidinium‐FSI^−^/TFSI^−^ electrolytes exhibit pronounced interphase degradation above 120°C–150°C, accompanied by intensified gas evolution and rapid interfacial resistance growth [172]. On high‐voltage cathodes, thermally accelerated oxidative decomposition of both IL constituents and previously formed CEI species becomes increasingly severe, leading to CEI thinning, impedance escalation, and exacerbated transition‐metal dissolution (Figure 6c) [177]. Collectively, these temperature‐driven interfacial instability ultimately constrains the durability and safety of ILEs‐based cells operated under thermally demanding conditions.

##### 3.2.1.3 Separator Compatibility

A further limitation at elevated temperatures arises from the thermal stability and electrolyte compatibility of separators. As mechanically delicate yet functionally indispensable components, separators must preserve electronic insulation while permitting unhindered ionic transport. However, conventional polyolefin separators such as polyethylene (PE) and polypropylene (PP) inherently soften and shrink upon heating. The built‐in thermal‐shutdown behavior of PE‐containing separators, typically activated near 120°C–135°C, causes the pore structure to collapse abruptly, blocking Li^+^ migration, which results in internal resistance sharp increase [76, 178]. While this shutdown effect affords short‐term protection in flammable carbonate‐based systems, it becomes counterproductive in nonvolatile ILE‐based cells that are otherwise capable of operating at elevated temperatures. Moreover, prolonged exposure to high‐temperature environments renders polyolefin separators susceptible to thermal deformation and oxidative degradation (Figure 6d) [179, 180]. Their intrinsically low surface energy further leads to poor wettability toward many ILs, resulting in insufficient electrolyte uptake, reduced interfacial contact, and compromised ionic conductivity under thermal stress [128, 181].

##### 3.2.1.4 Device‐Level Implications

The above‐mentioned multiscale degradation processes ultimately propagate to the device level, manifesting as gas accumulation, internal pressure buildup, increase of internal resistance, loss of electrode mechanical integrity, and compatibility issues with polymer separators that may undergo swelling or chemical attack in reactive IL environments at HTs. Taken together, these phenomena highlight that HT failure in ILE‐based systems is not governed by a single catastrophic event, but rather by a cascade of interconnected temperature‐dependent processes spanning bulk structure evolution, solvation chemistry, interphase dynamics, and electrochemical side reactions.

#### 3.2.2 Material Design Approaches

The diverse HT degradation pathways described above clearly demonstrate that stabilizing ILEs at elevated temperatures necessitates coordinated control over molecular architecture, solvation chemistry, and interfacial reactions. Therefore, effective mitigation strategies must extend beyond simple cation/anion selection and instead integrate multilevel design principles aimed at regulating reaction pathways and suppressing thermally activated instabilities.

##### 3.2.2.1 Targeted Ion Design

Rather than broad structural modification, HT electrolyte development increasingly emphasizes targeted ion design, wherein specific structural motifs are introduced to suppress dominant degradation pathways. On the cationic side, thermally robust frameworks such as phosphonium, pyridinium, and pyrrolidinium have demonstrated superior resistance to ring‐opening reactions and β‐H elimination at elevated temperatures, as evidenced by phosphonium ILs exhibiting thermal stability in the range of 200°C–275°C [182]. On the anion side, mitigating fragmentation susceptibility remains a critical challenge, particularly for fluorinated sulfonyl imide species such as FSI^−^ and TFSI^−^. To address this limitation, several strategies have been proposed, including the introduction of sterically hindered perfluoroalkanesulfonyl anions, modulation of electron‐withdrawing substituents, and partial replacement of labile S─N and S─F bonds with more thermally robust structural motifs [174]. For example, Weng et al. proposed a novel single‐solvent dual anion ILE comprising LiFSI and EMITFSI, which exhibits thermal stability up to 250°C. The synergistic interaction between FSI^−^ and TFSI^−^ promotes the formation of a robust SEI, thereby enabling dendrite‐free lithium deposition and highly reversible cathode electrochemistry (Figure 7a) [167]. Besides, our previous work designed a dual‐anion ILEs (0.8Pyr_14_FSI‐0.2LiTFSI) offering significantly enhanced stability of CEI through synergistic effect of FSI^−^ and TFSI^−^. In this system, FSI^−^ decomposes to form LiF‐rich interphases that suppress electrolyte side reactions and Ni^4+^‐driven degradation, while TFSI^−^ enhances the oxidation stability, together yielding a robust interphase and effectively inhibiting microcrack generation (Figure 7b) [183]. Such targeted anion engineering not only improves thermal decomposition thresholds but also reduces the formation of reactive intermediates that catalyze interfacial degradation and transition‐metal dissolution at HTs.

**FIGURE 7:**
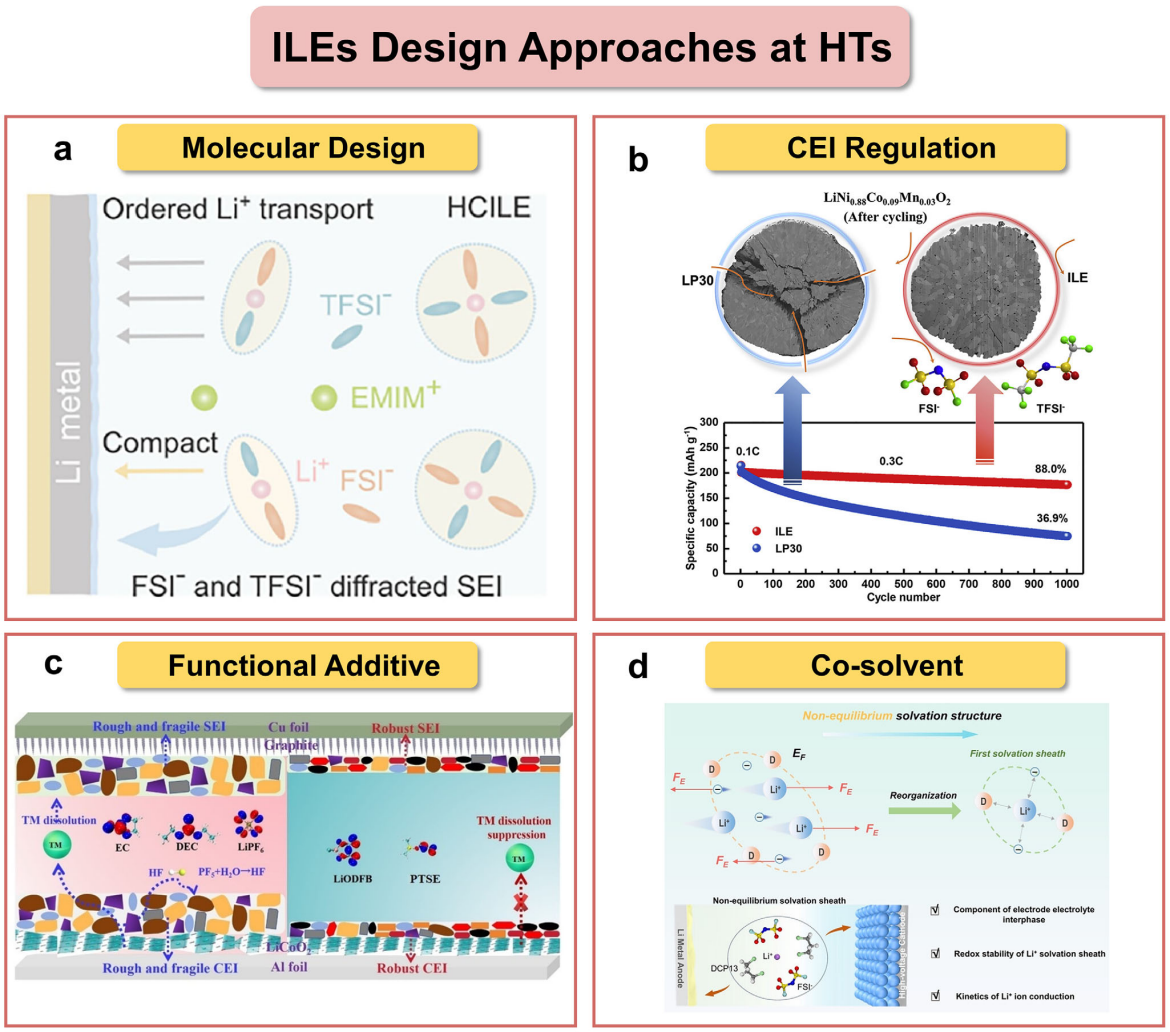
(a) Schematics of Li deposition from HCILE [167]. Reprinted with permission from ref [167]. Copyright 2024 Energy Storage Materials; (b) Dual anions strategy successfully regulates the formation of a stable CEI [183]. Reprinted with permission from ref [183]. Copyright 2021 Joule; (c) Mechanism of HT electrolyte on the performance of LIBs at elevated temperatures [184]. Reprinted with permission from ref [184]. Copyright 2024 Angewandte Chemie International Edition; (d) Schematic of the modulation of chloroalkane diluents on the solvate structure of lithium ions in locally concentrated ILEs [185]. Reprinted with permission from ref [185]. Copyright 2024 Angewandte Chemie International Edition.

##### 3.2.2.2 Interphase Engineering

At elevated temperatures, interfacial degradation becomes a dominant factor for cell failure in IL‐based electrolytes; consequently, the construction of thermally resilient SEI and CEI layers is indispensable. Although intrinsic ion design improves bulk electrolyte stability, sustained HT operation relies heavily on functional additives that preferentially decompose to generate inorganic‐rich interphases [186]. Additives capable of forming LiF, Li_2_O, Li_3_N, or phosphate‐rich interphases have proven particularly effective [174], as these inorganic products exhibit high thermal stability, low solubility, and strong resistance to interphase dissolution and structural reconstruction. By enabling preferential decomposition, such additives (e.g., lithium difluoro(oxalato)borate (LiDFOB)) redirect HT reaction pathways toward the formation of benign, protective surface films rather than reactive intermediates that accelerate electrolyte breakdown or transition‐metal dissolution (Figure 7c) [184, 187]. Despite their chemical diversity, effective additives possess several unifying characteristics: low LUMO energy levels, the ability to form compact inorganic layers, and strong suppression of parasitic reactions under oxidation and thermal stress. In addition, the co‐solvent strategy has also been employed in high‐temperature ILEs. Some flame‐retardant co‐solvents can enhance the regulation of the solvation structure, which is beneficial to the formation of stable SEI/CEI layers (Figure 7d). Therefore, these principles underline a unified interphase‐engineering strategy in which stabilizing decomposition pathways represents the most reliable route for improving the HT performance of ILEs [188].

##### 3.2.2.3 HT‐Resistant Separator Fabrication

Beyond electrolyte and interphase engineering, the development of thermally resilient separators is essential for fully exploiting the intrinsic HT advantages of ILEs. Conventional polyolefin separators suffer from severe thermal shrinkage, limited thermal resistance, and poor wettability, which undermine cell integrity long before the intrinsic limits of ILEs are reached [181]. Recent efforts have therefore focused on designing separators with enhanced thermal endurance and improved electrolyte affinity. For example, electrospun polytetrafluoroethylene (PTFE)/ polyvinylpyrrolidone (PVP) composite nanofibers synergistically combine the exceptional thermal stability of PTFE with the favorable surface affinity imparted by PVP, exhibiting negligible mass loss below 250°C, which highlights their suitability for HT applications [189]. Polyimide‐based architectures, such as polyimide (PI)/poly(ionic liquid)s (PILs) hybrid separators, also demonstrate remarkable dimensional stability from 60°C to 160°C and maintain structural integrity up to 400°C, while simultaneously achieving significantly higher electrolyte uptake compared with PP membranes [190]. Similarly, PIL‐derived nanofiber separators fabricated from poly(diallyldimethyl)ammonium bis(trifluoromethane sulfonyl)imide maintain morphological integrity at 150°C, exhibit a decomposition temperature near 400°C, and display excellent wettability toward ILEs, as evidenced by a low contact angle of 21° and exceptionally high electrolyte absorption capacity [191]. These advances clearly demonstrate that separator innovation is critical for unlocking the intrinsic thermal resilience of ILEs and enabling next‐generation battery systems capable of operation under extreme temperature conditions.

To better compare the major approaches for improving the high‐temperature performance of ILEs, their primary advantages and limitations are summarized in Table 3. As discussed above, high‐temperature failure in ILEs does not stem from a single factor, but rather from the coupled effects of bulk structural reorganization, interphase instability, and separator incompatibility. Therefore, effective high‐temperature electrolyte design requires not only intrinsically thermally robust ionic structures, but also stable interphases and compatible cell components.

**TABLE 3:** Material design approaches for improving the high‐temperature stability of ILEs.

Approaches	Subcategory	Advantages	Limitations
Targeted ion design	Heat‐resistant cations	High thermal stability; suppressed cation degradation	Limited control of interfacial instability
	Robust/dual‐anion design	High decomposition threshold; stable interphase	Anion fragmentation at extreme heat
Interphase engineering	Film‐forming additives	Stable interphase; suppressed side reactions	Additive consumption during long‐term cycling
	Flame‐retardant co‐solvents	Regulated solvation; improved SEI/CEI stability	Formulation sensitivity
HT‐resistant separators	Thermally stable polymer separators	High dimensional stability; good wettability	Limited mechanical robustness; interfacial mismatch

#### 3.2.3 Thermal Safety of Batteries

Unlike conventional carbonate‐based electrolytes, whose thermal failure is dominated by solvent volatilization, flammable gas evolution, and highly exothermic decomposition reactions, ILEs intrinsically redefine the thermal safety paradigm of rechargeable batteries at the molecular level. This fundamental advantage originates from their negligible vapor pressure, inherent non‐flammability, and high thermal decomposition thresholds, which collectively suppress the formation of combustible intermediates and delay the onset of thermal runaway [192]. However, the safety of ILE‐based batteries not only depends on electrolytes; it must be synergistically realized through optimized cell design, robust separators or supports, and controlled charge‐discharge management.

From a molecular design perspective, both cationic and anionic architectures play decisive roles in governing the thermal response of ILEs. Structural asymmetry, charge delocalization, and the incorporation of thermally robust functional groups effectively weaken long‐range ionic ordering and suppress crystallization, while simultaneously elevating decomposition temperatures. For example, the modified ILs demonstrate significantly enhanced flame retardancy alongside maintained or even improved electrochemical performance when intrinsically non‐flammable phosphate groups are built into IL structures [193]. Such molecular features not only enhance thermal stability but also fundamentally suppress flammable gas generation, thereby decoupling electrolyte decomposition from catastrophic thermal escalation. As a result, the transition from electrolyte degradation to self‐accelerating thermal runaway is substantially delayed, even under aggressive heating or overcharge scenarios.

Beyond intrinsic molecular stability, the incorporation of flame‐retardant co‐solvents and functional additives provides a clearly distinguishable safety lever, complementary to the inherent advantages of ILEs. Phosphorus‐ or fluorine‐containing flame retardants, when combined with ILEs, can further suppress ignition propensity and reduce heat‐release rates without compromising electrochemical stability [194]. Wang et al. developed a difluorinated IL‐based double‐salt electrolyte that integrates a difluorinated IL with LiTFSI/LiDFOB and dimethyl carbonate (DMC) co‐solvents. The high F/H ratio yields intrinsic non‐flammability, while thermogravimetric analysis confirms thermal stability exceeding 360°C. Importantly, cells employing this electrolyte successfully withstood stringent hot‐box (130°C), overcharge (5.16 V), and nail‐penetration tests without triggering thermal runaway, demonstrating that rational anion design can endow ILEs with not only superior HT tolerance but also robust resistance to multiple abuse scenarios [195]. Importantly, such additives function predominantly under abuse conditions, ensuring that thermal stabilization is activated precisely when the system approaches its failure threshold. This strategy enables a clear conceptual separation from prior discussions on performance optimization, highlighting thermal safety as an independent and designable electrolyte attribute.

While molecular and formulation‐level strategies establish the foundation for intrinsic thermal safety, the ultimate resistance to catastrophic failure emerges from system‐level integration. Recent advances demonstrate that coupling ILEs with thermally robust separators, such as ceramic‐reinforced or metal–organic framework–modified membranes, effectively suppresses thermal shrinkage and inhibits internal short‐circuit propagation [22]. Furthermore, semi‐solid or quasi‐solid ILE configurations restrict electrolyte mobility and suppress leakage under mechanical deformation, providing an additional barrier against thermal escalation [196].

These design principles become particularly critical in large‐format and high‐capacity battery systems, where thermal gradients, local hot spots, and mechanical stresses are significantly amplified. Under severe abuse conditions, including nail penetration, compression, and cutting, ILE‐based systems consistently exhibit reduced temperature rise, delayed voltage collapse, and the absence of ignition or explosion. Such behavior underscores a key distinction between thermal stability and thermal safety: even when electrochemical functionality is irreversibly compromised, ILE‐enabled batteries can maintain structural integrity and avoid catastrophic outcomes [34].

Collectively, these studies highlight that thermal safety in ILE‐based batteries is not a passive consequence of chemical robustness, but rather an emergent property arising from multiscale design, spanning molecular structures, electrolyte formulations, interphases, separators, and cell architectures. Future progress will require quantitative evaluation metrics beyond conventional cycling tests, including heat‐release kinetics, runaway onset temperatures, and abuse tolerance under coupled thermal‐mechanical‐electrical stresses.

### 3.3 Challenges and Strategies of IL‐Based Post‐Lithium Batteries

The preceding sections have mainly discussed ILEs in lithium‐based batteries, where their temperature‐dependent ion transport, phase behavior, and interfacial chemistry have been identified as the central factors governing electrochemical performance under extreme conditions. In fact, the application of ILEs is not limited to LIBs. Owing to their excellent intrinsic physicochemical properties, ILEs also hold great promise for non‐lithium battery systems, including Na‐, K‐, Mg‐, Al‐, and Zn‐based batteries. These cell chemistries offer distinct advantages in terms of resource abundance, cost, and sustainability, making them attractive alternatives or complements to lithium‐based energy‐storage technologies. Nevertheless, when operated under LTs or HTs, IL‐based nonlithium batteries face not only the common challenges discussed above, but also more system‐specific obstacles. In particular, the bulk ion conduction and the distinct plating/stripping behavior of alkali‐metal anodes necessitate greater caution in the design of ILEs [10]. Therefore, it is necessary to discuss the temperature‐dependent challenges and corresponding strategies of ILEs according to different nonlithium battery chemistries.

#### 3.3.1 Other Monovalent Batteries at Extreme Temperatures

Among non‐lithium battery systems, sodium‐ion batteries (SIBs) have received particular attention owing to the natural abundance, low cost, and favorable electrochemical characteristics of Na. As the sixth most abundant element in the Earth's crust and the lightest alkali metal next to Li, Na provides a sustainable charge carrier for large‐scale energy storage while retaining a relatively low redox potential and acceptable cell voltage [197]. However, simply transferring the electrolyte chemistry of LIBs to SIBs is not always effective. In conventional organic electrolytes, severe solvent decomposition, unstable SEI formation, and low CE often occur because the solvation structure, interphase chemistry, and Na^+^ migration behavior differ substantially from those of LIBs. In this regard, ILEs offer an attractive alternative [18]. Their solvent‐free ionic environment can reduce parasitic solvent reactions, which favors the formation of compact interphases and improves cycling reversibility. In addition, the weaker solvation of Na^+^ generally lowers the desolvation barrier, which partly explains why SIBs can exhibit more tolerant LT behavior than LIBs [198].

Despite these advantages, ILEs in SIBs still suffer from pronounced transport and interfacial limitations under extreme temperatures. At LTs, increased viscosity and enhanced ion association suppress bulk Na^+^ diffusion, while sluggish interfacial charge transfer leads to severe polarization and heterogeneous SEI growth. These issues can be alleviated through strategies similar to those developed for LIBs, such as weakly solvating co‐solvents and SEI‐forming additives [199]. Nevertheless, the selection of co‐solvents in Na‐based ILEs should be carefully balanced, as the SEI is highly sensitive to changes in electrolyte composition. Co‐solvents with insufficient reductive or oxidative stability may intensify parasitic interfacial reactions [10].

At HTs, Na‐based ILEs show a more complicated behavior. Elevated temperature decreases viscosity and enhances Na^+^ transport, which can improve electrode kinetics and even activate electrode materials that are sluggish at room temperature. However, the low melting point of Na metal (98°C) imposes a practical limitation before many IL components reach their intrinsic decomposition temperature [200]. Once the Na metal anode softens or approaches melting, the stability of the SEI and the uniformity of Na plating/stripping become difficult to maintain. Moreover, charged electrodes can accelerate the cathodic or anodic decomposition of ILE components at elevated temperatures, resulting in interphase reconstruction and impedance growth. To mitigate these HT limitations, one effective strategy is to exploit thermally stable highly concentrated FSI‐based ILEs while operating the cell in a controlled intermediate‐temperature region, where the transport and interfacial kinetics can be enhanced without triggering severe electrolyte decomposition. For example, Na_2_FeP_2_O_7_ coupled with NaFSI‐Pyr_13_FSI delivered a markedly improved rate capability at 90°C, reaching 83 mAh g^−1^ at 2000 mA g^−1^, far exceeding the value obtained at room temperature. More importantly, this system retained 93% of its capacity after 1500 cycles with an average CE of 99.9%, demonstrating that highly concentrated ILEs can effectively suppress continuous interfacial degradation while benefiting from thermally accelerated Na^+^ transport [201].

Another representative approach is to optimize the salt concentration and electrode architecture simultaneously. In carbon‐coated Na_3_V_2_(PO_4_)_3_ cells using NaFSI‐EMIFSI electrolytes, increasing the NaFSI fraction improved the high‐temperature rate capability by enhancing Na^+^ supply and decreasing interfacial resistance. And the long‐term cycling at 20C retained 89.2% of the initial capacity after 5000 cycles at 90°C [202]. Therefore, HT Na‐based ILE design should integrate electrolyte formulation with electrode interface engineering, rather than relying solely on the intrinsic thermal stability of ILs.

Potassium‐ion batteries (PIBs) represent another important monovalent system in which ILEs are highly valuable. K is abundant and inexpensive, and its ion, K^+^, usually exhibits weaker Lewis acidity and a smaller solvated radius than Li^+^ and Na^+^, which can facilitate rapid ion transport and reduce desolvation resistance. Nevertheless, PIBs exhibit interfacial behaviors distinct from both Li‐ and Na‐based systems at HTs. Owing to the larger ionic radius of K^+^, SEI components in PIBs are generally expected to show high solubility in polar electrolytes; however, K^+^ can also readily precipitate with large anions, making the construction of less‐soluble SEI more accessible than in Na systems [203]. Thus, compared to the co‐solvent strategy, the additive approach is more effective in this case. Sun et al. reported a highly innovative KCl‐buffered AlCl_3_/1‐ethyl‐3‐methylimidazolium chloride (EMICl) IL via the strategic incorporation of ethylaluminum dichloride (EtAlCl_2_) and KFSI as pivotal additives. Benefiting from high ionic conductivity and intrinsic nonflammability, this electrolyte generated robust K‐, Al‐, F‐, and Cl‐containing passivation layers, enabling safe and reversible K‐metal battery operation [204].

Overall, monovalent non‐lithium systems such as Na‐ and K‐based batteries exhibit several kinetic advantages at LTs. Owing to their weaker solvation interactions and generally lower desolvation barriers compared with Li^+^, Na^+^ and K^+^ can, in principle, support more favorable interfacial ion‐transfer kinetics at LTs. However, this advantage does not directly translate into stable wide‐temperature operation. At HTs, the highly reactive alkali‐metal/electrolyte interface becomes increasingly unstable, accompanied by accelerated SEI dissolution, reconstruction, and parasitic electrolyte decomposition. In this context, SEI‐stabilizing additives are particularly important, as they can promote the formation of thermally robust SEI, which can suppress continuous side reactions and maintain reversible metal plating/stripping in extreme temperature conditions.

#### 3.3.2 Multivalent Metal Batteries at Extreme Temperatures

Compared with monovalent alkali‐metal batteries, multivalent metal batteries exhibit fundamentally different temperature‐dependent limitations and opportunities. Mg, Zn, and Al can deliver multi‐electron redox reactions and high volumetric capacities, while the anodes generally possess higher thermal tolerance than Li, Na, and K [205]. More importantly, the deposition of multivalent metals is often less susceptible to severe dendritic growth because each deposited ion transfers multiple electrons and the corresponding deposition kinetics are typically more uniform. As a result, multivalent systems inherently offer improved fast‐charging capability and enhanced resistance to dendrite‐induced short circuits compared with many monovalent metal batteries [206, 207]. However, the high charge density of Mg^2+^, Zn^2+^, and Al^3+^ also leads to strong Coulombic interactions with solvents, anions, and interfacial species. Consequently, their electrochemical performance is often limited not by dendrite growth alone, but by sluggish bulk diffusion, slow charge‐transfer kinetics, and the formation of ion‐blocking passivation layers. These issues become particularly severe at LTs, where strong ion association and sluggish desolvation kinetics become the dominant barriers to efficient ion transport [208].

For Mg‐based and Zn‐based batteries, the strong coordination between divalent cations and surrounding ILs generates relatively high desolvation barriers, making interfacial charge transfer much more difficult than in Li‐, Na‐, or K‐based batteries. Consequently, LT ILE design often relies on strongly coordinating solvents or additives to facilitate cation dissociation and transport, which contrasts with the weak‐solvation strategies frequently adopted in monovalent systems. Another important distinction lies in interphase chemistry. While inorganic‐rich SEIs are generally desirable for alkali‐metal batteries, many inorganic decomposition products (MgF_2_, MgO, or Zn‐containing passivation compounds) exhibit poor multivalent‐ion conductivity [209]. Therefore, Mg and Zn batteries often benefit from organic‐rich or hybrid organic‐inorganic interphases that remain permeable to divalent ions while suppressing parasitic reactions. For Mg batteries, Ha et al. demonstrated reversible Mg plating/stripping in mixed oligoether‐Mg(TFSI)_2_ ILEs (diethylene glycol dimethyl ether (diglyme, G2) 1 : 56 mole ratio) [210]. However, many of the works in Mg batteries use aqueous solvents or non‐aqueous solvents, which are not compatible with Mg anodes, thus limiting these electrolytes to fundamental studies rather than practical application. For Zn batteries, Hawkins et al. reported a deep‐eutectic electrolyte composed of Zn(TFSI)_2_, EMITFSI, and γ‐butyrolactone, which optimized viscosity, ionic conductivity, and Zn^2+^ diffusion by regulating contact ion pairs and ion aggregates. This electrolyte enabled reversible Zn electrodeposition down to −60°C [211]. In another representative study, Yu et al. proposed an ionic liquid “water pocket” strategy by introducing water‐immiscible EMIFSI into aqueous Zn electrolytes. The ionic liquid environment enveloped H_2_O‐dominated Zn^2+^ solvates, suppressed water activity, and regulated Zn deposition through the synergistic roles of EMI^+^ and FSI^−^, leading to a uniform inorganic‐rich SEI and stable Zn_0.25_V_2_O_5_·nH_2_O||Zn cycling even at 60°C [212].

Al batteries are distinct from both monovalent and other multivalent systems because metallic Al is covered by a dense native Al_2_O_3_ layer that blocks direct Al deposition and dissolution. As a result, electrolyte design is centered on controlling chloroaluminate speciation rather than conventional SEI engineering. Chloroaluminate ILs, typically based on AlCl_3_/EMICl, are widely employed because AlCl_4_^−^ and Al_2_Cl_7_^−^ species can dissolve or reconstruct the oxide layer and enable reversible Al plating/stripping. Under extreme temperatures, however, the balance between oxide‐layer regulation, corrosion, and interfacial stability becomes increasingly critical. Pang et al. reported fast‐charging Al‐chalcogen batteries using a molten‐salt electrolyte with excellent resistance to dendritic shorting at HTs [213]. Meng et al. subsequently developed a quaternary molten‐salt electrolyte that enabled rapid‐charging Al─S batteries at 85°C while maintaining high‐rate performance [214].

Overall, ILEs in multivalent metal batteries require a design philosophy distinct from that of Li‐, Na‐, and K‐based systems. Although multivalent metals generally exhibit superior dendrite resistance and fast‐charging potential, their high charge density introduces strong cation‐solvent and cation‐anion interactions that significantly increase desolvation barriers and interfacial transport resistance. Consequently, solvation regulation and the construction of ion‐conductive organic‐rich or hybrid interphases are often more important than maximizing inorganic SEI content. Future ILEs for multivalent batteries should combine efficient multivalent‐ion transport and thermally robust interphases to fully exploit the intrinsic high volumetric capacity of multivalent metal batteries under extreme temperature conditions.

## 4 Interfacial Chemistry and Electrode Compatibility

The stability of the interface between ILEs and electrodes is a decisive factor dictating the electrochemical performance and safety of energy storage systems operated across a wide temperature range [35]. Temperature fluctuations significantly impact interfacial reactions, ion transport, and SEI/CEI formation, which in turn may lead to performance degradation or even failure, depending on the electrolyte composition and electrode chemistry [22, 195]. As a consequence, elucidating these temperature‐dependent interfacial phenomena is indispensable for enabling the long‐term performance of ILE‐based batteries. This section delves into the distinctive interfacial behaviors at alkali‐metal anodes, high‐voltage cathodes, and solid‐state interfaces, with emphasis on mechanistic insights, molecular‐level design principles, and unresolved challenges that need to be addressed.

### 4.1 SEI Formation on Alkali Metal Anodes

The SEI layer plays a foundational role in dictating the stability, safety, and lifespans of batteries. Functioning as a passivation layer, it permits selective ion transport while inhibiting continuous electrolyte depletion and uncontrolled parasitic reactions [215]. In ILEs, the unique ionic environment, broad ESW, and distinctive solvation structures exert a pronounced influence on the nucleation and chemical composition and structural integrity of the SEI, especially under extreme temperature conditions [216].

#### 4.1.1 Challenges in Forming Stable SEI in ILEs

Despite the notable thermal and electrochemical stability of ILEs, establishing compact, ion‐conductive, and electronically insulating SEI on alkali metal surfaces like Li, Na, or K remains a persistent challenge [44]. A primary obstacle stems from the limited reducibility of common ILE anions, including TFSI^−^ and FSI^−^, which exhibit high reduction potentials (below 1.0 V vs. Li^+^/Li). As a result, interfacial reduction proceeds slowly and non‐uniformly, producing chemically heterogeneous and structurally discontinuous SEI layers [23].

In contrast to organic carbonate or ether electrolytes, where solvents’ molecule readily undergo reductive cleavage to yield a uniform Li_2_CO_3_‐rich SEI layer, ILEs lack neutral solvent molecules capable of homogeneous decomposition [23]. Consequently, the SEI formed in ILEs is typically dominated by inorganic constituents (e.g., LiF, Li_2_S_x_, or Li_3_N) but deficient in polymeric or organic components, which collectively imparts mechanical fragility and elevated interfacial resistance [217]. This deficiency becomes particularly pronounced at LTs, where ionic transport is suppressed, and reduction reactions proceed even more unevenly, which in turn aggravates dendritic deposition or “mosaic‐type” unstable interphases [218]. Furthermore, the high viscosity intrinsic to ILEs combined with strong cation‐anion interactions impedes uniform Li^+^ distribution at the electrode‐electrolyte interface, which magnifies electric‐field gradients and accelerates localized deposition instabilities [140, 219]. Such conditions manifest as heightened interfacial impedance and reduced CE, particularly below −20°C [220].

At elevated temperatures (> 80°C), the dominant degradation pathways shift. Improved ion mobility and thermal activation accelerate the fragmentation of both anions and cations, generating reactive intermediates such as sulfonyl radicals and carbon‐fluorine fragments [221]. These reactive species attack and corrode the SEI, leading to continuous reconstruction and progressive loss of mechanical integrity [222]. Indeed, reversible SEI formation and decomposition cycles have been observed in IL‐based LMBs after long‐term operation at HTs, reflecting the thermodynamic sensitivity of the SEI [223].

These temperature‐dependent SEI degradation mechanisms are consistent with the broader interfacial perspective summarized by Jamil et al., who emphasized that the evolution of metal‐anode interphases in ILEs is governed not only by the intrinsic electrolyte chemistry, but also by operational parameters, including electrolyte composition, salt concentration, temperature, and current density [10]. Taken together, these observations demonstrate that although ILEs exhibit impressive intrinsic thermal resilience, their interfacial electrochemistry remains complex and highly sensitive to the interplay of ion association, reduction selectivity, and morphological evolution. Therefore, developing next‐generation ILs capable of engineering controlled, adaptive, and self‐healing SEI represents a critical frontier for ensuring stable performance across a broad temperature range.

#### 4.1.2 SEI Layer Design Strategies

The chemical composition and structural characteristics of ILs critically determine the chemistry, morphology, and stability of the SEI [150]. Functional additives have been employed as an alternative strategy for precisely tuning and engineering SEI characteristics [219]. Furthermore, a co‐solvent strategy can also tune SEI formation under a certain design of solvation structure [19].

Among commonly used anions, FSI^−^ generally undergoes more facile reductive fragmentation, yielding LiF‐rich interphases that are compact and ionically conductive [221]. TFSI^−^, in contrast, exhibits a higher resistance to decomposition, leading to a reduced proportion of LiF in the decomposition products and a greater prevalence of more complex compounds, such as Li_2_S_2_O_4_, LiSO_2_CF_3_ and Li_2_NSO_2_CF_3_. Although less prone to direct reductive decomposition, cations significantly affect interfacial structure via solvation structure and EDL modulation [165]. For instance, imidazolium‐based ILs exhibit a pronounced adsorption tendency on negatively polarized electrode surfaces, driven by π–π interactions and cation‐metal interactions. This adsorption leads to the formation of dense EDL structures that may impede the lithium‐ion desolvation and the uniform development of SEI. On the other hand, asymmetric or sterically bulky cations are known to reduce ion‐pairing association, enhancing Li^+^ mobility near the electrode interface and suppressing dendritic nucleation, which is particularly beneficial for LT operation [138]. So, the role of cations in the EDL is crucial for the formation of a compact and uniform SEI. For example, monofluorinated piperidinium (MFP_13_^+^) cations preferentially reside at the SEI surface, exhibiting moderate adsorption on the lithium metal surface and effective shielding effects. More importantly, MFP_13_^+^ efficiently attracts fluorine‐containing anions into the EDL, thereby preventing the undesirable transition of the lithium metal anode interface from an anion‐rich to an anion‐deficient structure, with the cycle life of the battery greatly improved (Figure 8a) [224].

**FIGURE 8:**
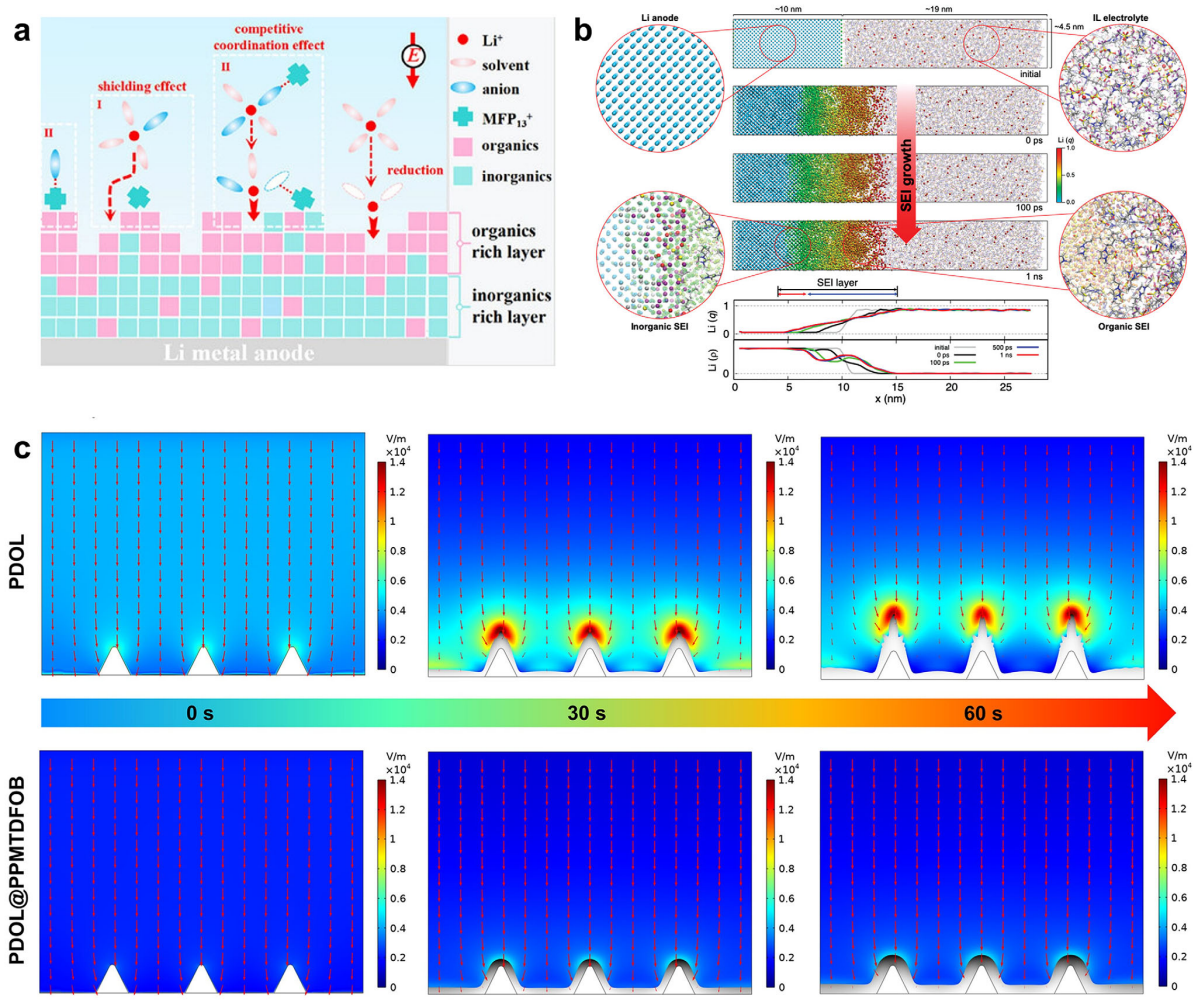
(a) A possible SEI model and regulation of interfacial chemistry via monofluorinated cations [224]. Reprinted with permission from ref [224]. Copyright 2026 Advanced Materials; (b) the formation of the SEI layer from the simulation of BMIMTFSI ILE [225]. Reprinted with permission from ref [225]. Copyright 2022 Advanced Energy Materials; (c) evolution snapshots at different time steps from the phase‐field simulation of electric field intensity within poly (1,3‐dioxolane) (PDOL) and PDOL@1‐methyl‐1‐[(methylthio)methyl]piperidin‐1‐ium difluoro(oxalato)borate (PPMTDFOB) cells [226]. Reprinted with permission from ref [226]. Copyright 2025 Science Advances.

To further manipulate SEI composition, numerous studies have explored functional additives such as LiNO_3_ [227], fluorinated ethers [228], or inorganic halides [161]. These compounds undergo preferential reduction to form Li_3_N or LiF‐enriched SEI, enhancing SEI homogeneity and suppressing continuous IL decomposition. Similarly, carbonate co‐solvents can themselves function as SEI‐forming additives, generating robust interphases on the electrode surface that protect against reductive decomposition of the IL component [88]. Such hybrid electrolytes typically yield hierarchically structured SEI, which integrates an inorganic inner layer for ion transport and a flexible organic outer layer for stress accommodation (Figure 8b) [225].

Another critical consideration is the temperature‐dependent evolution of SEI morphology in IL‐based electrolyte systems. At LTs, the interplay of sluggish interfacial kinetics and elevated electrolyte viscosity generally results in the formation of heterogeneous, particulate SEI layers [137]. Conversely, elevated temperatures promote the development of denser and more coherent interphases, albeit with associated risks of over‐reduction and structural reorganization (Figure 8c) [226]. To achieve consistent performance over a wide temperature range, the SEI in ILEs should be maintained as thin, compact, and uniform, which is essential for minimizing the propensity for uncontrolled lithium dendrite growth.

### 4.2 Cathode Electrolyte Interphases

On the cathode side, the stability of ILEs is governed not only by their intrinsic anodic stability, but more critically by the chemistry, morphology, and transport characteristics of the CEI formed under high potentials. Conventional carbonate electrolytes undergo severe oxidative decomposition above ∼4.3 V, leading to uncontrolled CEI thickening, impedance rise, and accelerated capacity decay [226]. Many ILEs offer wider ESWs (often > 5 V), which makes them appealing for high‐voltage cathodes and wide‐temperature operation [34]. Nevertheless, anodic stability alone is not sufficient: under elevated temperatures or prolonged cycling, oxidative fragmentation of IL constituents can still occur, continuously reshaping the CEI, aggravating surface degradation, and promoting the loss of active materials [229].

A defining advantage of ILEs is the alterability of CEI formation through cation and anion engineering. Fluorinated anions (e.g., TFSI^−^ and FSI^−^) often favor the formation of inorganic‐rich CEI that contains LiF or metal fluorides, which are beneficial for suppressing further electrolyte oxidation and mitigating transition‐metal dissolution. Meanwhile, oxidative resilience can be enhanced by selecting more robust cation families (notably phosphonium or sulfonium cations) and pairing them with oxidation‐tolerant anions, thereby lowering the tendency for parasitic oxidation and enabling thinner, more uniform CEI layers [13]. Complementarily, functional additives and passivation promoters, like LiNO_3_ [230], borates [231], can introduce sacrificial or preferentially oxidized species that promote rapid CEI establishment [232]. It is worth noting that although the present discussion is mainly centered on lithium‐based systems, a similar CEI‐regulation concept has also been demonstrated in potassium‐based batteries, where ILE engineering enables stable interphases under high‐voltage operation, consistent with the design logic illustrated in Figure 9a,b [233].

**FIGURE 9:**
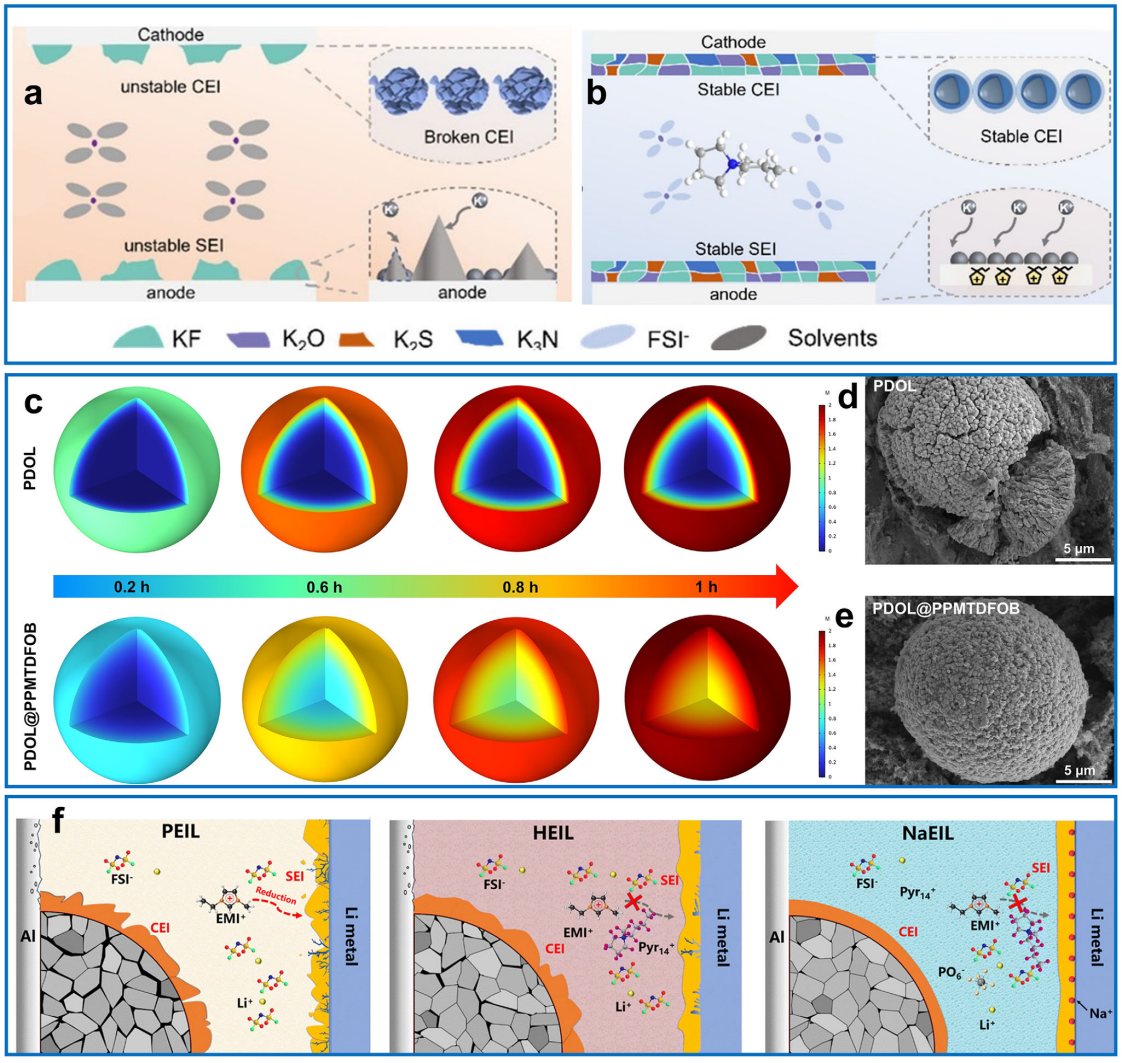
Schematic diagram of the effects of (a) KFSI/Pyr_13_FSI and (b) KFSI/DME and KPF_6_/EC:DMC:EMC (4:3:2 by volume) electrolytes in PBs, respectively [233]. Reprinted with permission from ref [233]. Copyright 2025 ACS Energy Letters; c) Finite element analysis simulating the lithiation behavior of PDOL and PDOL@PPMTDFOB with NCM811 cathode at the current density of 1 C; SEM images of cycled NCM811 cathode with PDOL (d) and PDOL@PPMTDFOB electrolytes(e) [226]. Reprinted with permission from ref [226]. Copyright 2025 Science Advances; f) Schematic of the effects of PEIL, HEIL and NaEIL electrolytes on the secondary cathode particles in NCM83||Li cells [234]. Reprinted with permission from ref [234]. Copyright 2025 Energy & Environmental Science.

Importantly, the optimal CEI is strongly temperature dependent, and the requirements at LTs and HTs are not necessarily congruent. At LTs, cathode polarization is markedly exacerbated by sluggish interface kinetics in conjunction with limited electrolyte transport; therefore, CEI must remain sufficiently thin and ionically percolating, while also accommodating mechanical mismatch induced by contraction without the formation of ion‐blocking cracks [34]. Under such circumstances, a CEI enriched with organic or polymeric components, offering improved mechanical compliance and crack tolerance, can be advantageous, provided that the associated increase in interfacial resistance remains limited and does not compromise charge‐transfer kinetics [156]. By contrast, at elevated temperatures, oxidative parasitic reactions are significantly accelerated, and interphase dissolution‐reconstruction processes become more pronounced. Under these conditions, the CEI must exhibit superior chemical robustness and enhanced mechanical rigidity, with a higher inorganic fraction to mitigate transition metal dissolution, suppress oxygen‐driven surface reactions, and stabilize the cathode surface against sustained oxidative stress [16]. These divergent requirements imply that effective cathode protection across a wide temperature window may ultimately require graded or multilayer CEI architectures that combine an ionically conductive and mechanically compliant outer layer to ensure LT tolerance with an inorganic‐dominant inner barrier that provides HT durability and oxidative stability (Figure 9c–e).

Despite substantial recent progress, current strategies for CEI construction still face several intrinsic limitations. First, CEI formation in ILEs is often highly sensitive to cathode chemistry, upper cut‐off voltage, and trace impurities. As a result, the interphase composition can vary significantly across different cathode families and even cycling protocols, undermining reproducibility and transferability. Second, additive‐derived CEI may not be truly self‐limiting: continuous additive consumption during prolonged cycling can induce gradual interphase thickening, impedance growth, and concomitant modifications in cathode surface chemistry, particularly under HT conditions. Third, IL‐derived CEI can undergo dynamic reconstruction, including softening, partial dissolution, and re‐precipitation, causing intermittent exposure of reactive surface sites and promoting cumulative side reactions, which remain a key obstacle to achieving long‐term stability across wide temperature windows [232]. Finally, most reported strategies focus on either bulk electrolyte stability or initial CEI formation, whereas the evolution of CEI under coupled thermal‐electrochemical stress conditions remains insufficiently understood.

Looking forward, several research directions appear particularly promising for achieving durable, self‐limiting CEI compatible with wide‐temperature and high‐voltage operation. One viable approach is to tailor IL structures to bias oxidation toward benign pathways that rapidly generate dense, electronically insulating yet Li^+^‐permeable CEI components, thereby enforcing self‐passivation rather than continuous decomposition. Another approach is to develop synergistic, multi‐component designs that couple IL chemistry with interfacial promoters to create temperature‐adaptive CEI, either through intentionally graded compositions or through dynamic bonding motifs that preserve integrity upon thermal cycling (Figure 9f) [234]. In addition, cathode‐surface engineering (e.g., thin inorganic coatings or catalytically inert interlayers) can be combined with ILEs formulations to reduce the interfacial driving force for oxidation and to stabilize transition‐metal chemistry, which lowers CEI reactivity and improves long‐term reproducibility. Ultimately, advancing wide‐temperature ILEs cathode interfaces will require integrating molecular‐level electrolyte design with mechanistically informed interphase engineering to deliver CEI layers that are simultaneously ionically efficient, chemically resilient, and mechanically stable under both LT polarization and HT oxidative stress.

### 4.3 Temperature‐Adaptive SEI/CEI

The protective interphase (SEI/CEI) formed in batteries is often discussed at a specific temperature. For wide‐temperature batteries, however, such a static interphase is inherently insufficient. A desirable SEI or CEI should not only be chemically stable at a given temperature, but also maintain compatible ion transport, mechanical integrity, and regulated decomposition chemistry as the electrolyte structure evolves with temperature [235]. In this sense, temperature‐adaptive SEI/CEI refers to an interphase whose composition, structure, and ion‐transport characteristics can be dynamically regulated in response to thermal variations. Rather than serving as a passive and static barrier, such an interphase should preserve selective ion transport and electronic insulation while accommodating thermally induced changes in electrolyte mobility, electrode reactivity, and mechanical/chemical stability.

At LTs, the dominant problem is kinetic in nature. Increased viscosity, sluggish desolvation, and slow interfacial charge‐transfer processes can render even a chemically stable interphase detrimental if it becomes excessively dense and ion‐blocking. Therefore, the construction of ion‐conductive and mechanically compliant interphases is often more critical than simply maximizing the fraction of inorganic decomposition products, particularly in LT battery systems [236]. In contrast, interphase regulation at HTs is governed more strongly by thermodynamically driven degradation and parasitic reactions. Although faster ion transport is generally achieved, interphase dissolution, transition‐metal dissolution, oxygen release from high‐voltage cathodes, and electrolyte decomposition are all significantly accelerated [237]. Under these conditions, the SEI/CEI must function as a thermally robust kinetic barrier capable of suppressing continuous electrolyte consumption while retaining sufficient ionic permeability.

Among various electrolyte systems, ILEs provide a particularly attractive platform for engineering temperature‐adaptive interphases, as their ion‐rich environments enable interfacial chemistry to be tailored through cation‐anion coordination, ion aggregation, and EDL regulation. A representative example is the thermally adaptive, PVDF‐HFP/ILE membrane reported by Jabeen et al. In this system, PVDF‐HFP was integrated with 1‐ethyl‐1‐methylpyrrolidinium bis(trifluoromethylsulfonyl)imide to construct an adaptive electrolyte membrane for sodium‐metal batteries. Rather than relying only on the intrinsic thermal stability of the IL, the membrane regulates the electrolyte state and solvent mobility through a thermoresponsive quasi‐solid/gel transition. At HTs, the membrane behaves as a solid electrolyte that suppresses cathode/electrolyte side reactions and promotes the formation of a stable CEI, while the optimized PVDF‐HFP/IL coordination environment simultaneously contributes to the formation of a robust SEI on the Na‐metal anode [238]. This example indicates that temperature‐adaptive interphase design can be achieved by coupling the thermal response of the electrolyte phase with coordinated SEI/CEI formation at both electrodes.

The concept of adaptive interphase regulation has also been demonstrated in IL‐based aqueous Zn batteries. For example, EMIBF_4_ and hydrated Zn(BF_4_)_2_ were combined to form a eutectic ILE, in which the Zn^2+^ solvation shell was reconstructed into a BF_4_^−^‐rich coordination environment. This temperature‐resilient solvation structure simultaneously improves bulk electrolyte properties and interfacial stability. At LTs, reduced water connectivity and a weakened hydrogen‐bond network enhance the antifreezing capability of the electrolyte and facilitate Zn^2+^ desolvation. At HTs, decreased water activity suppresses hydrogen evolution, Zn corrosion, and by‐product accumulation. More importantly, adaptive interfacial protection is achieved through a dual‐shield mechanism at the Zn anode. The accumulated BF_4_^−^ species undergo interfacial decomposition to form a ZnF_2_‐rich static SEI, which serves as an ion‐permeable chemical barrier against continuous parasitic reactions. Meanwhile, positively charged EMIM^+^ species become enriched near the Zn surface and act as a dynamic electrostatic shield, homogenizing the local electric field and guiding uniform Zn deposition during cycling [239]. These findings highlight that temperature‐adaptive interphase design extends beyond the formation of a chemically stable SEI and instead relies on dynamic coupling among solvation regulation, interfacial chemistry, and electrostatic field modulation.

More broadly, recent studies on non‐IL temperature‐adaptive electrolytes have further shown that thermally responsive solvation structures can be achieved through deliberate regulation of anion‐related interactions. For instance, a 6.5 Ah pouch‐type lithium‐ion battery employing a temperature‐adaptive electrolyte exhibited stable operation across an exceptionally wide temperature range from −30°C to 130°C [240]. In addition, anion‐aggregated solvated electrolytes featuring temperature‐insensitive solvation structures have also been developed for LT lithium‐metal batteries [241]. Furthermore, by employing an anion‐regulated strategy distinct from conventional solvent‐dominated approaches to tailor both the solvation structure and EDL characteristics, Yuan et al. achieved excellent compatibility between ether‐based electrolytes and lithium metal over a wide temperature range from −60°C to 80°C simply by adjusting the ratio of different lithium salts [242]. Although these strategies have not been demonstrated in an IL system, they collectively underscore the critical role of anion‐mediated solvation engineering in achieving temperature‐adaptive interfacial chemistry and provide valuable design principles that may be readily extended to the highly tunable ILE platform. These studies further suggest that adaptive SEI/CEI design should be understood as a coupled process involving temperature‐dependent solvation structure, anion‐derived interphase chemistry, EDL reconstruction, and ion‐flux regulation, thereby providing valuable design principles for extending these concepts to the highly tunable ILE platform.

### 4.4 Interfaces in Solid Electrolytes

The integration of ILEs with solid or quasi‐solid electrolytes, including pseudo‐solid‐state configurations, has emerged as an effective approach to mitigate the inherent interfacial limitations of rigid solid‐state electrolytes and electrodes [243, 244]. The incorporation of ILEs improves interfacial wettability, diminishes contact resistance, and offers variable ionic routes, thus enhancing electrochemical performance over a broad temperature range [136]. In addition, previous reviews have suggested that the incorporation of ILs can facilitate the formation of stable and robust SEI layers, thereby mitigating electrolyte decomposition and suppressing dendrite growth in pseudo‐solid‐state electrolyte systems [244]. Liu et al. further noted that ILs can enhance ion conduction in all‐solid‐state battery electrolytes, underscoring their utility as functional transport regulators in solid and quasi‐solid electrolyte architectures [19]. Their inherent polarity and adjustable ion‐dipole interactions facilitate close contact between the electrode and solid host, while concurrently establishing ionically conductive yet chemically stable interphases that reduce parasitic reactions [37].

This interfacial advantage also underpins the development of IL‐containing gel polymer electrolytes (GPEs). Niu et al. summarized that ILs can serve as plasticizers in polymers such as poly(ethylene oxide) (PEO), poly(ethylene glycol) (PEG), poly(methyl methacrylate) (PMMA)–PEG, and PVDF‐HFP to enhance ion transport in LIBs [17]. Yet, conventional IL‐GPEs often suffer from limited electrochemical stability and mechanical robustness because of poor IL/polymer compatibility and cycling‐induced phase separation. In this context, poly(ionic liquid)s provide a more compatible host for ILs, while crosslinking, block/graft copolymerization, polymer incorporation, and inorganic fillers can further reinforce IL‐based GPEs. Moreover, incorporating EMITFSI into the Li_6.4_La_3_Zr_1.4_Ta_0.6_O_12_ (LLZTO) system not only enhances Li^+^ transport across grain boundaries but also mitigates interfacial degradation by buffering mechanical stress and chemical incompatibility, enabling stable battery operation over a wide temperature range of 20°C–80°C [245]. Sulfide‐based solid electrolytes possess ionic conductivities comparable to those of liquid electrolytes and maintain high ion transport capabilities across a wide temperature range; however, their poor solid–solid interfacial contact with electrodes remains a major challenge. Recently, novel ion‐conducting polymer binders prepared using ILEs have been proposed as a promising solution. For instance, a cathode fabricated with LiTFSI‐Pyr_14_TFSI as an additive directly matched with Li_10_SnP_2_S_12_ (LSPS) exhibits over 70% capacity retention after 80 cycles at 0.5 C rate [246]. However, the interfacial behavior of ILEs is highly temperature dependent. At LTs, partial vitrification of ILs components restricts segmental motion and slows Li^+^ migration, whereas at HTs, excessive ILs diffusion or decomposition may induce interphase reconstruction and impedance growth [247]. At present, numerous problems persist in the application of ILs to solid‐state batteries, including ion transport losses at LTs and interfacial interactions with active electrodes [19, 79]. Consequently, acquiring a more profound molecular‐level comprehension of the temperature‐dependent interfacial dynamics is essential for the systematic design of ILE‐based battery systems.

## 5 Conclusions and Outlook

Over the past decade, ILEs have emerged as a paradigm‐shifting material class capable of resolving persistent challenges of maintaining electrochemical performance stability over a wide temperature range. This review systematically examines the intricate interplay among structure, properties, and interfaces in ILEs, with particular emphasis on temperature‐dependent evolution of solvation structures, ion transport mechanisms, and interfacial chemistry at LTs and HTs. Through critical analysis of recent advancements, we further identify persistent challenges in extreme thermal environments and propose optimization frameworks spanning from molecular design to interphase engineering.

ILs exhibit negligible volatility, a broad electrochemical window, and tunable ion‐ion correlation dynamics. These attributes collectively position ILs as superior structural platforms for thermally adaptive electrolytes compared to conventional organic counterparts. Rational cation‐anion pairing strategies, side‐chain modification, and mixed IL formulations have been demonstrated to precisely modulate key parameters including viscosity, ionic conductivity, and solvation configuration. For instance, the introduction of molecular asymmetry or flexible functional groups disrupts ion ordering excessively, hence reducing the T_m_. Moreover, incorporating ILs into polymeric or inorganic frameworks yields solid‐like ILEs with improved dimensional stability and interfacial contact, offering a promising direction toward quasi‐solid or solid‐state structures.

From the perspective of ion transport dynamics and solvation chemistry, engineering the solvation environment emerges as a pivotal strategy for balancing LT fluidity and HT stability. The coordination number of Li^+^ exhibits solvent‐dependent variability, ranging from weakly coordinating ionic environments to heterogeneous mixed‐solvent matrices. This structural adaptability prevents cryogenic ion aggregation while suppressing thermal degradation pathways under elevated thermal conditions. HILEs that incorporate a small fraction of molecular solvents or gel matrices have demonstrated enhanced thermal stability by decoupling ionic mobility from structural relaxation. The fundamental requirement for preserving stable ionic conductivity across extended temperature ranges lies in maintaining dynamic ion transport networks paired with adaptable solvation cages capable of thermally responsive reconfiguration.

Interfacial chemistry is the most critical and challenging factor influencing the performance of ILE‐based devices in practical implementation. Establishing stable and ionically conductive SEI on alkali metal anodes remains challenging due to sluggish interface kinetics at LTs and side reactions at HTs. Research indicates that altering the ionic species, such as utilizing ILEs containing FSI^−^ to produce LiF‐rich and homogeneous SEI layers, significantly enhances the stability of the alkali metal/electrolyte interface. In addition, under prolonged HT exposure, divergent degradation pathways emerge: certain ILs undergo self‐limiting decomposition, whereas others experience continuous degradation at HTs, ultimately compromising cycling stability. Recent advancements in surface engineering, including interfacial additives and protective coatings, have shed light on strategies to balance these competing reactions and extend operational windows up to 150°C.

The development of IL‐based solid electrolytes has further bridged the gap between liquid‐like mobility and solid‐state stability. By infusing ILs into porous solid frameworks, researchers have realized hybrid electrolytes exhibiting simultaneous high ionic conductivity and mechanical robustness while keeping the non‐volatility even at very LTs. Within these systems, liquid domains facilitate ion migration across grain boundaries, while solid frameworks impart dendrite‐suppressing mechanical properties and thermal stability. Nonetheless, temperature‐dependent evolution of hybrid interfaces, particularly glass transition‐induced rigidity at subzero temperatures and excessive liquid diffusion under thermal stress, still poses challenges.

Prospectively, the realization of ILEs with extended operational temperature tolerance necessitates a multiscale engineering paradigm spanning molecular design, structural optimization, and interfacial regulation. Several directions are highlighted herein for future investigations:

1. Molecular‐level architectonics for wide‐temperature ILEs: The key to achieving outstanding wide‐temperature performance lies in thermoresponsive ILE systems featuring dynamic molecular adaptability. This includes introducing flexible alkyl functionalities to enhance LT segmental dynamics, and integrating fluorinated or aromatic moieties to strengthen HT oxidation resistance. Rationally designing asymmetric cations or multi‐anion architectures to simultaneously achieve strong Coulombic interactions and entropy‐driven ion dissociation, ensuring temperature‐resilient ion transport in a wide temperature range. Such molecular engineering can suppress crystallization and promote ion dissociation in cryogenic conditions, as well as control excessive structural loosening and parasitic interfacial reactions at HTs. Therefore, descriptiveness‐guided molecular architectonics can offer a more practical choice for balancing ion transport at LTs and chemical and structural stability at HTs with a wide operating window.
2. Engineering SEI and CEI: Given the interfacial dominance in ILEs performance, research should focus on establishing self‐equilibrating SEI/CEI with temperature‐modulated composition and kinetics. LiNO_3_ and FEC serve as prototypical IL‐compatible additives capable of thermally programmed decomposition to yield ionically conductive yet thermally stable (up to 200°C) interphases. In situ spectroscopic monitoring coupled with ab initio molecular dynamics simulations may unravel the dynamic equilibrium between interphase formation and electrolyte degradation.
3. Solvation Regulation of ILEs by Tailored ILs: A promising technical route is to introduce into ILEs the ion‐regulated solvation strategies that have recently emerged in non‐IL electrolytes, where tailored anion‐related interactions are used to modulate solvation sheaths and electric double layers across wide temperature ranges. For ILEs, this concept could be extended beyond simple anion regulation by leveraging their intrinsically designable ionic species and ultimately promoting the spontaneous formation of temperature‐adaptive interphases. In this way, ILEs may achieve intrinsic solvation reconfiguration without relying solely on conventional molecular co‐solvents. In practice, this strategy could be combined with the functional IL design, additive engineering, and co‐solvent regulation discussed above, thereby enabling synergistic tuning of solvation structures, ion transport, and interfacial chemistry, consequently achieving superior electrochemical performance at both LTs and HTs.
4. Microemulsion‐ or suspension‐type ILEs: Another promising direction is to move beyond conventional homogeneous ILEs and deliberately construct microemulsion‐ or suspension‐type ILEs with tailored multiphase structures. In these cases, dispersed domains, interfacial regions, and continuous IL phases could potentially provide extra degrees of freedom for ion transport, solvation regimes, and interfacial chemistry in cold temperatures. At LTs, rationally designed microheterogeneous structures may suppress crystallization, buffer viscosity increase, and preserve percolated ion‐transport pathways by decoupling local mobility from bulk structural freezing. At HTs, thermally robust IL‐rich continuous phases may help confine reactive components, redistribute interfacial ion populations, and mitigate thermally accelerated side reactions. However, it is still time to control phase stability, sedimentability, component compatibility, and reproducibility at large temperature levels. Future work should therefore focus on coupling colloidal/interfacial design with IL chemistry to develop structurally adaptive ILEs that remain transport‐efficient and interfacially stable from cryogenic to HT conditions.
5. Intelligent screening and data‐driven design: The enormous compositional diversity of ILs demands high‐throughput screening strategies. Machine learning and artificial intelligence can accelerate the identification of optimal ion combinations by correlating properties (e.g., polarity, symmetry, viscosity, and phase transition) with temperature‐dependent performance. Creating comprehensive databases of laboratory‐tested ILEs systems will allow researchers to forecast their stability for thermal and electrochemical factors. This will significantly reduce the necessity for trial‐and‐error methodologies.
6. Practical feasibility and scalability: Finally, the successful deployment of ILEs in commercial batteries requires balancing performance with manufacturability and safety. Large‐scale synthesis of ILs with reduced cost and environmental impact, along with compatibility with existing cell assembly processes, will be essential for technological translation. Moreover, establishing standardized evaluation protocols for wide‐temperature testing will facilitate fair benchmarking across research groups and accelerate industrial adoption. Particularly, overcoming the high‐viscosity‐related challenges in electrolyte filling, porous‐electrode wetting, and lean‐electrolyte operation, while maintaining compatibility with current separators, binders, and cell fabrication processes, will be crucial for the practical implementation of ILEs.

In summary, ILEs are crucial in rendering energy storage technologies functional throughout diverse climates. Through meticulous molecular engineering, strategic interface management, and data‐driven design, it is feasible to construct ILEs that exhibit exceptional ionic conductivity, extensive electrochemical stability, and compatibility with various materials across a temperature range of −50°C to 150°C. The ongoing integration of materials science, electrochemistry, and computational intelligence will undoubtedly transition ILEs from the laboratory to practical applications. Increased research and interdisciplinary collaboration may result in ILE‐based batteries that will power the future of electric vehicles, space exploration, and energy systems in extreme conditions, achieving unprecedented safety and reliability.

## Author Contributions

**En Xie**: writing – original draft, investigation. **Chengdong Liu**: writing – original draft, investigation. **Xinghao Wang**: writing – original draft, investigation. **Jian Wang**: writing – review and editing. **Zhen Chen**: writing – review and editing. **Huihua Li**: writing – review and editing. **Wenlong Cai**: writing – review and editing. **Hao Li**: writing – review and editing. **Yang Peng**: writing – original draft. **Fanglin Wu**: writing – original draft, writing – review and editing, conceptualization, resources. **Stefano Passerini**: writing – review and editing, resources, conceptualization. **Haolin Tang**: writing – review and editing, resources, conceptualization.

## Conflicts of Interest

The authors declare no conflict of interest.

## Data Availability

Data sharing not applicable to this article as no datasets were generated or analysed during the current study.
